# Fully 4D-Printed Near-Infrared-Actuated Lab-on-Valve Solid-Phase Extraction Devices

**DOI:** 10.1021/acs.analchem.4c05363

**Published:** 2024-12-25

**Authors:** Chia-Hsun Kuo, Cheng-Kuan Su

**Affiliations:** Department of Chemistry, National Chung Hsing University, Taichung City 402202, Taiwan, ROC

## Abstract

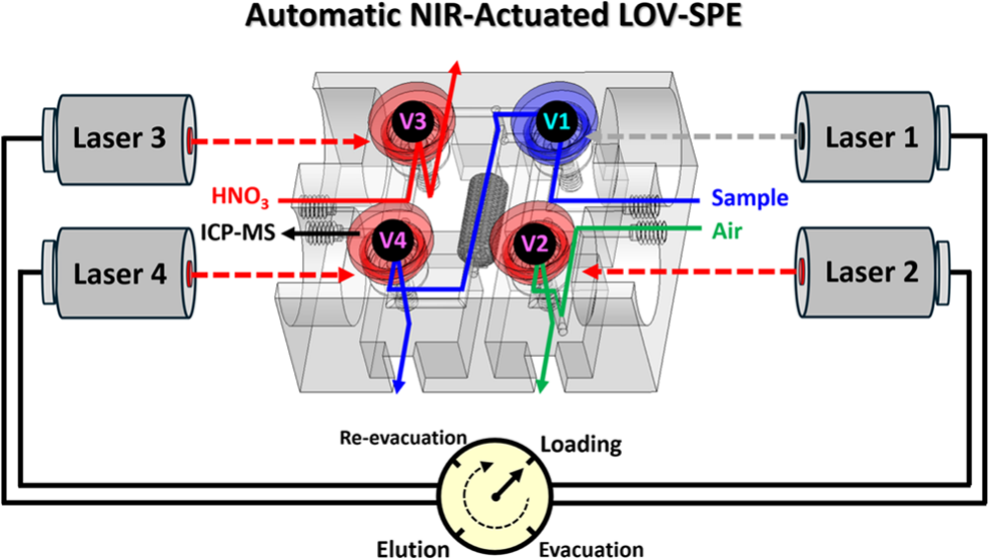

Four-dimensional printing (4DP) technologies can expand the functionality of stimuli-responsive devices to enable the integration of multiple stimuli-responsive parts into a compact device. Herein, we used digital light processing three-dimensional printing technique, flexible photocurable resins, and photocurable resins of the temperature-responsive hydrogels comprising *N*-isopropylacrylamide (NIPAM), *N*,*N*′-methylenebis(acrylamide) (MBA), and graphene for 4DP of a lab-on-valve (LOV) solid-phase extraction (SPE) device. This device featured flow manifolds and a monolithic packing connected by four near-infrared (NIR)-actuated temperature-responsive switching valves composed of a poly(NIPAM/MBA) (PNM) ball pushing a flexible membrane. NIR irradiation caused the deswelling of the PNM ball [temperature > volume phase transition temperature (VPTT) of the hydrogel], and the valve was opened to switch the flow direction. The termination of this irradiation caused the swelling of the PNM ball (temperature < VPTT of the hydrogel) to close the valve and thus recover the original flow direction to achieve the automatic NIR-actuated fluid control. The optimized 4D-printed NIR-actuated LOV-SPE device enabled a fully automatic SPE scheme coupled with inductively coupled plasma mass spectrometry for the determination of Mn, Co, Ni, Cu, Zn, Cd, and Pb ions (detection limits = 0.1,6.8 ng L^–1^). The reliability of this analytical method was validated by determining the metal ions in the four reference materials (CASS-6, SLRS-5, 1643f, and Trace Elements Urine L-2) and environmental water and human urine samples. Our results demonstrated the capability and applicability of 4DP technologies for advancing the automation of LOV-SPE schemes and related analytical methods.

## Introduction

Owing to its simplicity, cost-effectiveness, and high efficiency, the solid-phase extraction (SPE) technique, which uses selective sorbents to enrich target analytes and eliminate sample matrices, is routinely used to improve the reliability of conventional atomic spectrometric methods [e.g., inductively coupled plasma mass spectrometry (ICP-MS), inductively coupled plasma optical emission spectrometry] and their applicability for trace metal analysis.^1−4^ To automatize an SPE scheme in closed analytical systems using the lab-on-valve (LOV) technique coupled with flow injection/sequential injection analysis (FIA/SIA) can substantially minimize trace metal contamination and eliminate the errors introduced by manual operation.^2,5−7^ However, the needs of expensive valve apparatuses (e.g., switching valves, solenoid valves, selectors, and control systems) and the experience of valve arrangement in flow manifolds could restrict the usability of FIA-LOV systems for handling stepwise procedures in general laboratories. Alternatively, stimuli-responsive devices that can respond to stimulus and show corresponding shape programming for fluid control have drawn considerable attentions, as exemplified by magnetically actuated valves,^8,9^ thermoresponsive valves,^10,11^ pH-responsive valves,^12,13^ humidity-responsive valves,^14,15^ and light-driven valves.^16,17^ Nonetheless, the fabrication of stimuli-responsive valves using conventional methods usually involves labor-intensive and time-consuming processes, such as molding, alignment, bonding, and postfabrication treatments, which makes these valves merely applicable to only simple flow switching.^18−21^

In the past decade, three-dimensional printing (3DP) technologies have been utilized to customize a variety of analytical devices in digitally controlled and automatic processes.^22−26^ In terms of fluid control, 3D-printed devices, such as membrane-based valves,^27−30^ injection valves,^31−33^ slope valve,^34^ and Quake-style valve,^35^ have been illustrated. However, additional mechanical driving units (e.g., pneumatic pumps, driving flows) are necessary to facilitate their actuation processes,^27−29,32,35^ which makes 3D-printed valves suitable only for special laboratorial procedures. On the other hand, although many 3D-printed SPE devices are better suited for trace metal analysis than commercial SPE columns, commercial valve apparatuses and control units are still required to manipulate the sample, rinsing solution, and eluent streams for establishing the corresponding automatic LOV-SPE analytical systems.^36−47^ The use of stimuli-responsive materials for the 3DP-based fabrication and integration of multiple functional stimuli-responsive parts in compact analytical devices should expand the applicability of 3D-printed devices to advance conventional analytical schemes.

Four-dimensional printing (4DP) technologies based on the use of conventional 3DP with stimuli-responsive materials along or with nonresponsive materials are effective in developing stimuli-responsive devices that can display stimuli-induced shape programming and thus realize complex time-dependent geometric functions.^48−51^ Although 4DP technologies can be used to directly fabricate stimuli-responsive devices, to the best of our knowledge, fluid control based on the programming of 4D-printed stimuli-responsive valves for the automation of a LOV-SPE scheme has never been reported. Hence, new fabrication strategies allowing one to reduce the complexity and costs of developing the FIA-LOV devices which integrate stimuli-responsive valves, flow manifolds, and SPE columns and enabling sensitive and reliable trace metal analysis in complex real samples are highly sought after.

Inspired by the above demand, this study demonstrates that 4DP technologies can be used to fabricate programmable stimuli-responsive devices for fluid control and automation of an SPE scheme and thus achieve reliable and accurate trace metal analysis. We used the print-pause-print digital light processing (DLP) 3DP technique, flexible photocurable resins, and photocurable resins of the temperature-responsive hydrogels comprising *N*-isopropylacrylamide (NIPAM), *N*,*N*′-methylenebis(acrylamide) (MBA), and graphene to fabricate an SPE device. This device featured a monolithic packing connected by four near-infrared (NIR)-actuated temperature-responsive switching valves composed of a poly(NIPAM/MBA) (PNM) ball that pushed a flexible membrane covering a T-shaped channel. The dehydration-induced deswelling of the PNM ball upon NIR irradiation [temperature > volume phase transition temperature (VPTT) of the PNM hydrogel] resulted in the valve opening to switch the flow direction. When this irradiation was terminated, the hydration-induced swelling of the PNM ball (temperature < VPTT of the PNM hydrogel) resulted in the valve closing to restore the original flow direction.^52−58^ The 4DP-based fabrication of the NIR-actuated temperature-responsive switching valves allowed us to tune the valve response time by adjusting the composition of the PNM ball, valve design, and laser actuation conditions and realize programmable NIR-actuated fluid control and, hence, automation of an SPE scheme. After the optimization of the (i) design and fabrication of the NIR-actuated temperature-responsive switching valves, (ii) valve actuation programming, and (iii) extraction conditions for the monolithic packing, the 4D-printed NIR-actuated LOV-SPE device enabled a fully automatic SPE scheme for coupling with ICP-MS for the sensitive and reliable determination of Mn, Co, Ni, Cu, Zn, Cd, and Pb ions. Furthermore, we compared the analytical performance of this system with that of a commercial automatic LOV-SPE system comprising a 3D-printed SPE column and three electric switching valves and validated the reliability and applicability of this analytical method by applying it to determine the metal ions in four reference materials and environmental water and human urine samples.

## Experimental Section

### Chemicals

NIPAM (415324), MBA (146072), diphenyl(2,4,6-trimethylbenzoyl)phosphine oxide (TPO; 415952), and disodium hydrogen phosphate (Na_2_HPO_4_; 255793, trace metals basis) were purchased from Sigma-Aldrich. Graphene nanoplatelets (453160500, 2–10 nm; lot number: A0446371) were purchased from Thermo Fisher Scientific. Nitric acid (HNO_3_; 6901, ultrapure grade) was purchased from J.T. Baker. Sodium hydroxide (NaOH; 106466, Suprapur grade) was purchased from Merck. Water purified using a Milli-Q IQ 7000 water purification system (Merck Millipore) was used to prepare all of the solutions. Working solutions were prepared by the serial dilution of multielement calibration standards containing Mn(II), Co(II), Ni(II), Cu(II), Zn(II), Cd(II), and Pb(II) (10 mg L^–1^; N9300233, PerkinElmer) with 10 mM phosphate buffer [with pH adjusted using 5.0% HNO_3_ (v/v) and 5.0% NaOH (w/v) solutions]. Photocurable resins of the temperature-responsive hydrogels were prepared by mixing NIPAM, MBA (cross-linker), graphene, and TPO (photoinitiator) in ethanol (Figure S1A) to fabricate the responsive part. Flex 57A flexible resins [QTS Corporation; comprising tri(propylene glycol) diacrylate, 1,6-hexanediol diacrylate, acrylate oligomers, and TPO; Figure S1B] and Aqua Clear resins (Phrozen; comprising bisphenol A ethoxylate dimethacrylate, 4-acryloylmorpholine, and TPO; Figure S1C) were used to fabricate nonresponsive parts.

### 4DP of NIR-Actuated LOV-SPE Device

**Figure 1:**
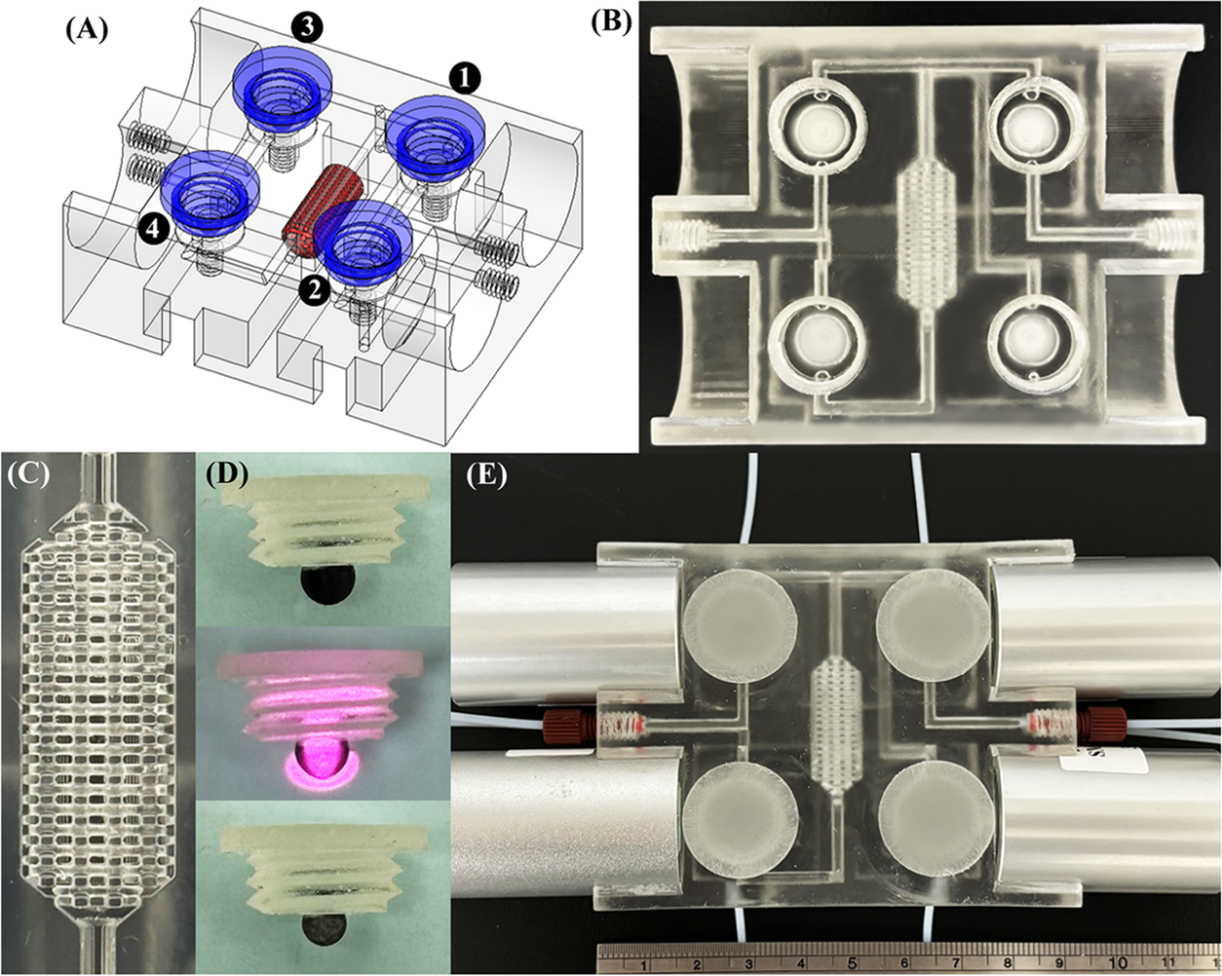
(A) Computer-aided design (CAD) drawing of the NIR-actuated LOV-SPE device, containing flow manifolds (gray part), a monolithic packing (red part), and four NIR-actuated temperature-responsive valves (blue parts). Photographs of the (B) base with the monolithic packing and four valve seats with the flexible membrane at the bottom of the valve seat, (C) monolithic packing stacked by the interlacing cuboids, (D) valve cover with the PNM ball before (top), during (middle), and after (bottom) NIR irradiation, and (E) assembled device fitted with eight flat-bottom male connectors, each with a piece of PTFE tubing, and four fiber collimators.

The SPE device featured one base and four demountable valve covers (Figures 1A and S2). The base (Figure 1B) included flow manifolds, eight fittings for a standard 10–32 flat-bottom male connector (three loading ports, one ICP-MS inlet port, and four waste ports), a monolithic packing stacked by interlacing cuboids arranged layer by layer with a 90° twist angle and featuring a cone-shaped design at both ends (Figure 1C), four valve seats, and four holders at the sides for fiber collimators. The valve cover (Figure 1D) contained a PNM ball attached to (designed as a part inserted into) the center of the cover end. The NIR-actuated temperature-responsive valve comprised a PNM ball that pushed a flexible membrane covering a T-shaped channel (thumbtack-shaped flow chamber) with a fixed distance (3.8 mm) from the cover end to the flexible membrane in the valve seat. Finger-tight screw thread pairs between the valve cover and seat enabled the alignment of the PNM ball, flexible membrane, and T-shaped channel. Detailed device dimensions are shown in Figure S2. Prior to NIR irradiation (Figure 1D, top), the hydration-induced swelling of the PNM ball [at room temperature (25 °C); < VPTT of the PNM hydrogel] pushed the flexible membrane to close the bottom channel (waste port) and guide the stream to flow through the horizontal channel (flow chamber). When the PNM ball absorbed thermal energy during continuous NIR irradiation (Figure 1D, middle and bottom), the dehydration-induced deswelling of the PNM ball (temperature > VPTT of the PNM hydrogel) resulted in the recovery of the flexible membrane and made the stream to flow into the bottom channel (waste port).

The base and valve covers were modeled using the SolidWorks 2020 (Dassault Systèmes) computer-aided design (CAD) software and fabricated using a DLP 3D printer (Phrozen Sonic Mini 8K; curing time of 10 s and *z*-axis resolution of 50 μm) operated based on the print-pause-print technique (fabrication time: 253 min for base and 83 min for valve cover; weight: 124.3 g for base and 2.1 g for valve cover; total material cost: US$ 6.18). The Flex 57A flexible resins were used to fabricate the whole base, with the flexible membranes covering the T-shaped channels as valve seats. The Aqua Clear resins were used to fabricate the valve cover until the end of the male screw thread (nonresponsive part); then, the photocurable resins of the graphene-incorporated temperature-responsive hydrogels were used to continue the fabrication of the PNM ball and allow its appropriate attachment to the center of the cover end (Figure 1D). After fabrication, the four valve covers were fitted to the corresponding valve seats. Eight flat-bottom male connectors (P-840 and P-844, IDEX Health & Science) with a short piece of polytetrafluoroethylene (PTFE) tubing (inner diameter of 0.03 in.) were fitted. The assembled SPE device was washed with a solution of 0.5% (v/v) HNO_3_ for at least 12 h to remove uncured resins and metal contaminants. A four-channel fiber-coupled laser system (FC-808, CNI Laser; 808 nm) equipped with fibers (T021-Z) and collimators (FA10866; spot sizes: 1.0, 3.0, and 5.0 mm at 10 cm) and controlled by the Laser Device Control software (v. 1.0) were used to program the NIR actuation of the four valves. Prior to performing experiments, we inserted four fiber collimators into the four holders to align the light beams with the PNM balls (Figure 1E).

### Methods and Apparatus

**Figure 2:**
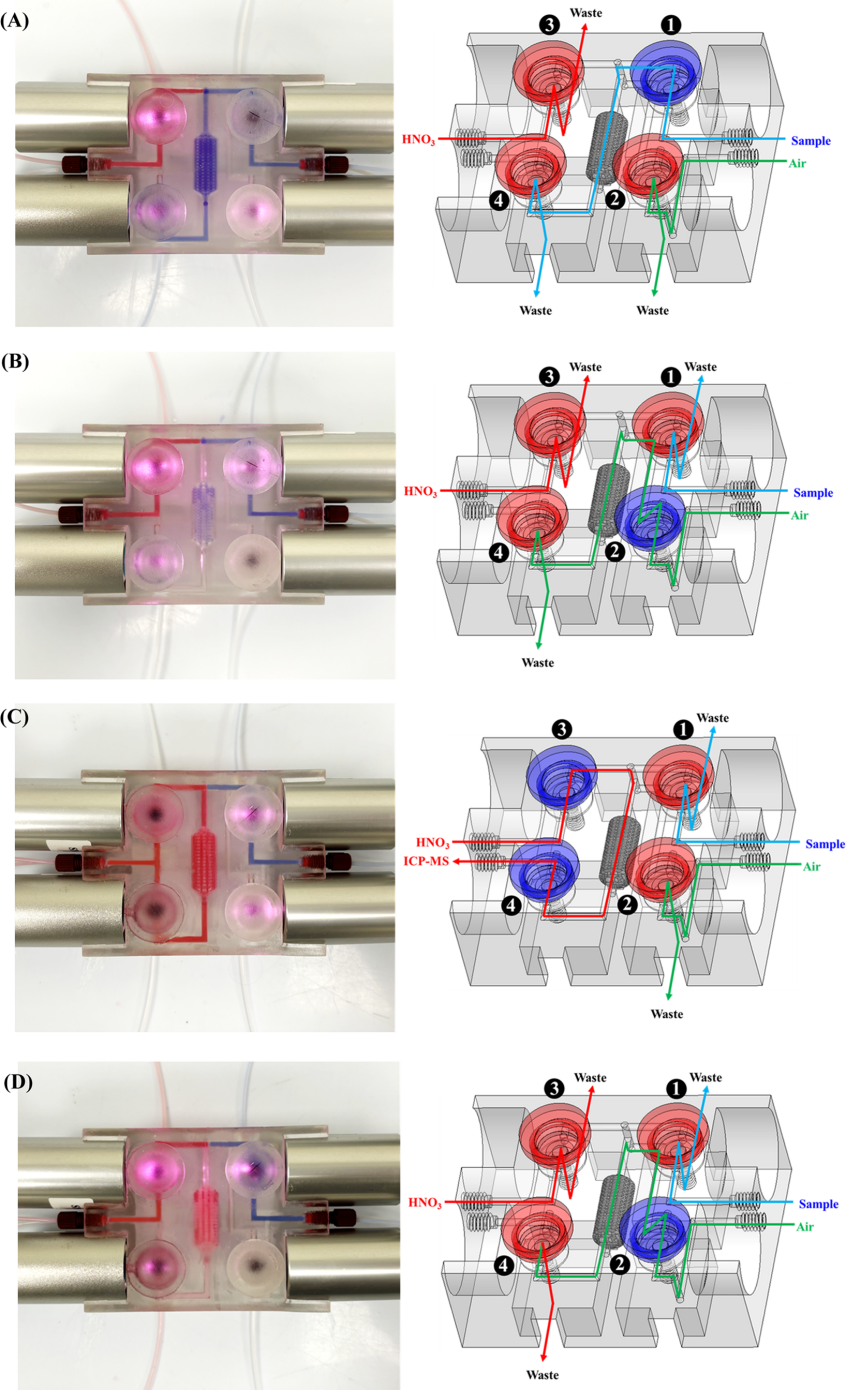
Photographs of the SPE scheme performed using the 4D-printed NIR-actuated LOV-SPE device. (A) Sample loading: loading of the conditioned sample (blue) into the monolithic packing for the extraction of the metal ions through the loading port of Valve 1 and removal of sample matrices through the waste port of Valve 4 (NIR irradiation of Valves 2–4; Valve 1 opened/Valve 2 closed/Valve 3 closed/Valve 4 closed). (B) Evacuation: loading of the air stream (transparent) through the loading port of Valve 2 to remove the sample matrices through the waste port of Valve 4 (NIR irradiation of Valves 1, 3, and 4; Valve 1 closed/Valve 2 opened/Valve 3 closed/Valve 4 closed). (C) Elution: loading of the eluent (red) through the loading port of Valve 3 to elute the extracted metal ions from the monolithic packing and transport the eluted metal ions into the ICP-MS system through the inlet port of Valve 4 (NIR irradiation of Valves 1 and 2; Valve 1 closed/Valve 2 closed/Valve 3 opened/Valve 4 opened). (D) Re-evacuation: loading of the air stream (transparent) through the loading port of Valve 2 to evacuate the residual eluent for loading of the next sample (NIR irradiation of Valves 1, 3, and 4; Valve 1 closed/Valve 2 opened/Valve 3 closed/Valve 4 closed). Blue arrow: sample; red arrow: eluent; green arrow: air stream.

The SPE scheme, including sample loading, evacuation, elution, and re-evacuation steps, was automatized (Table S1, Figures 2 and S3) by programming the NIR-actuated temperature-responsive switching valves. These four valves were used to control the sample stream (Valve 1), air stream for evacuation (Valve 2), eluent stream (Valve 3), and transportation of the eluted metal ions into the ICP-MS instrument (Valve 4). In the sample loading step, the conditioned sample (pH 8.0) was loaded into the monolithic packing (flow rate: 1.0 mL min^–1^; loading time: 85 s; loading volume: 1.4 mL) through the loading port of Valve 1 using a peristaltic pump (Miniplus 3, Gilson) to extract the metal ions, while the residual sample matrices were removed through the waste port of Valve 4 (Figure 2A; NIR irradiation of Valves 2–4). In the evacuation step, an air stream from Valve 2 was used to evacuate the monolithic packing (flow rate: 1.0 mL min^–1^; loading time: 85 s; evacuation volume: 1.4 mL) and completely remove the sample matrices through the waste port of Valve 4 (Figure 2B; NIR irradiation of Valves 1, 3, and 4). Then, an eluent stream from Valve 3 was used to elute the extracted metal ions from the monolithic packing (elution flow rate: 1.0 mL min^–1^; elution time: 245 s; elution volume: 4.1 mL) and transport them into the ICP-MS system (Agilent 7700x, Agilent Technologies) through the inlet port of Valve 4 for the time-resolved analysis (integration time: 50 ms) of the elution peak areas at *m*/*z* 55 (Mn), 59 (Co), 60 (Ni), 64 (Zn), 65 (Cu), 114 (Cd), and 208 (Pb) for external calibration (Figure 2C; NIR irradiation of Valves 1 and 2). After analysis of the metal ions, the air stream from Valve 2 was again used to evacuate the residual eluent (flow rate: 1.0 mL min^–1^; loading time: 85 s; evacuation volume: 1.4 mL) for loading of the next sample (Figure 2D; NIR irradiation of Valves 1, 3, and 4). The four valves were programmed to change their position at the designed time point of the SPE procedure and maintain the position until the step was finished. To compare the performance of the 4D-printed NIR-actuated LOV-SPE device and its analogue using commercial valves, a LOV-SPE system was developed by combining a 3D-printed SPE column with the same monolithic packing fabricated using the Flex 57A resins, three eight-port electric switching valves [C22Z-3188, Valco; programmed using a laptop and a serial valve interface (SIV-110, Valco)], a peristaltic pump, and the an ICP-MS instrument (Table S2 and Figure S4).

### Sample Preparation and Real Sample Analysis

The developed method was applied to the analysis of the metal ions in the four reference materials [1643f (fresh water; National Institute of Standards and Technology), SLRS-5 (untreated river water; National Research Council of Canada), CASS-6 (nearshore seawater; National Research Council of Canada), and Seronorm Trace Elements Urine L-2 (human urine, SERO)]. Seawater, river water, groundwater, municipal wastewater, and human urine samples, collected by in situ filtration using 0.45 μm syringe filters (CHROMAFIL Xtra H-PTFE, Macherey-Nagel) and acidification (0.5% HNO_3_, v/v), and these spiked with the target metal ions (0.05 μg L^–1^ for Co, Cd, and Pb; 0.5 μg L^–1^ for Mn, Ni, Cu, and Zn) were analyzed. All samples were neutralized to pH 8.0 using 10 mM phosphate buffer (e.g., pH 12.6 for 1643f; pH 11.5 for acidified seawater) and analyzed without further treatment. Statistical comparisons were performed using Student’s two-tailed unpaired *t*-test.

## Results and Discussion

### 4D-Printed NIR-Actuated LOV-SPE Device

For automation of a LOV-SPE scheme without using commercial valve apparatuses, we employed the print-pause-print DLP 3DP technique with temperature-responsive materials to fabricate an SPE device featuring four NIR-actuated temperature-responsive switching valves to control the passage of the sample, air stream (evacuation), and eluent through an SPE monolithic packing. We evaluated the effects of the (i) composition of the photocurable resins used to fabricate the temperature-responsive PNM ball, (ii) valve design, and (iii) laser actuation conditions on the valve response times to optimize the automation of an SPE scheme in the designed time sequence.

**Figure 3:**
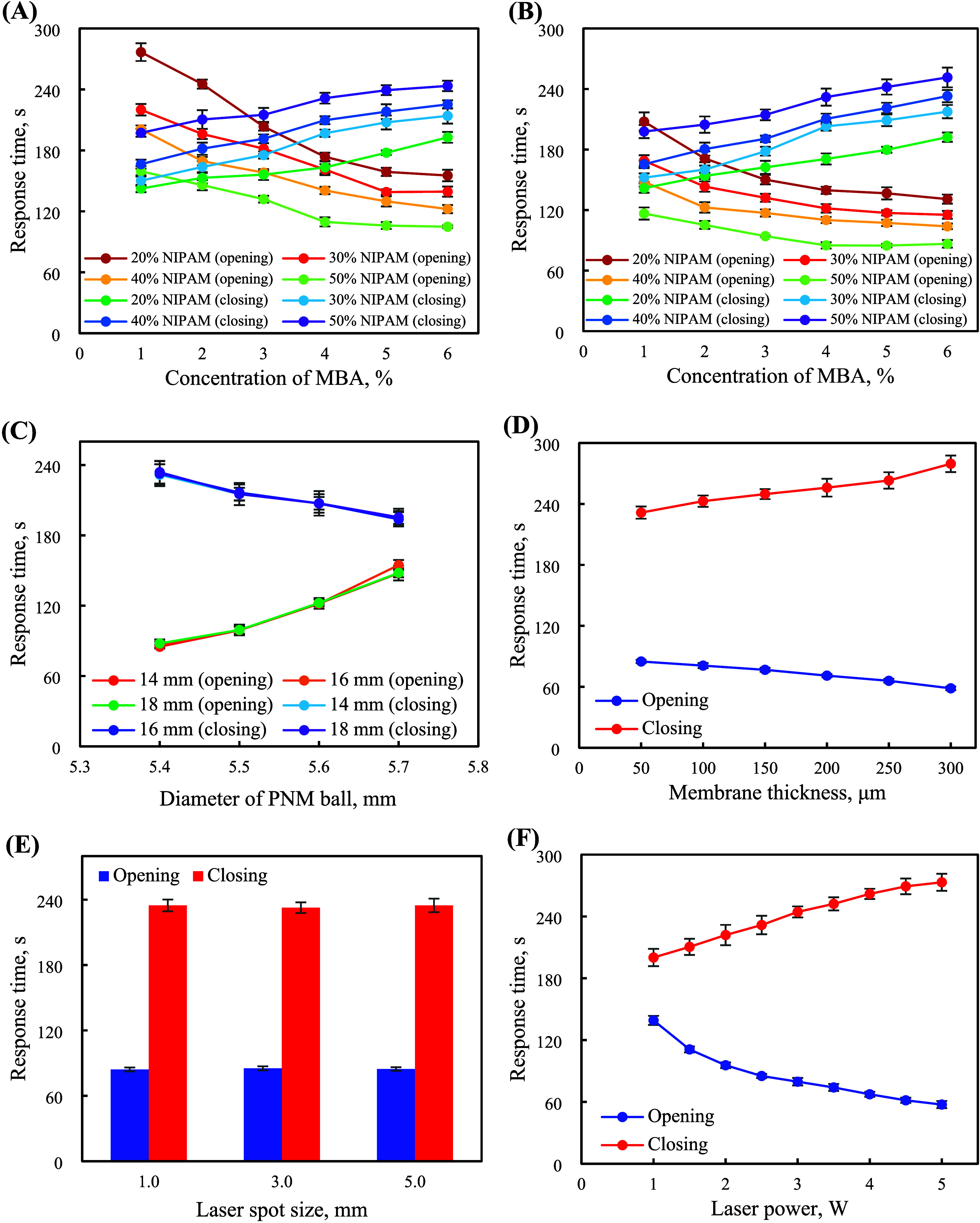
Measured valve response time plotted with respect to the (A) contents of NIPAM and MBA (with 1.0% graphene, w/v), (B) contents of NIPAM and MBA (with 2.0% graphene, w/v), (C) diameters of the PNM ball and the flexible membrane, (D) thickness of the flexible membrane, (E) laser spot size, and (F) laser power. The valve response time was measured as the time interval required for switching the flow from horizontal to vertical direction (valve opening) after NIR irradiation or switching the flow from vertical to horizontal direction (valve closing) after terminating the NIR source. Error bars represent standard deviations (*n* = 8).

The incorporation of graphene nanoplatelets substantially increased the NIR absorption efficiency due to their broadband light absorption and strong light-to-heat conversion abilities but did not affect the VPTT of the PNM hydrogel (27.7 °C; Figure S5).^52−59^Figures 3A,B and S6A show that the response time of the switching valves was determined by the PNM (de)swelling kinetics. The valve opening time (from 85 ± 2 to 347 ± 10 s) decreased with the increasing NIPAM (20 → 50%, w/v), MBA (1.0 → 6.0%, w/v), and graphene (0.5 → 2.0%, w/v) contents of the photocurable resins because of the increase in (i) the PNM hydrophobicity and curing density (with the increasing NIPAM and MBA contents), which favored the evacuation of water from the hydrogel^60^ for its deswelling, and (ii) NIR absorption efficiency (with the increasing graphene content; Figure S6B). The valve closing time (from 142 ± 9 to 252 ± 10 s) increased with the increasing NIPAM (20 → 50%, w/v) and MBA (1.0 → 6.0%, w/v) contents because of the concomitant increase in curing density and, hence, the amount of thermal energy that had to be dissipated for swelling of the PNM ball.

At fixed NIPAM (50%, w/v), MBA (4.0%, w/v), and graphene (2.0%, w/v) contents (VPTT: 27.7 °C; Figure S5) and a distance of 3.8 mm from the cover end to the flexible membrane in the valve seat, the valve opening time increased with the increasing diameter of the PNM ball (5.4 → 5.7 mm, designed as a part inserted into the cover end at a distance of 1.6–1.9 mm), presumably because of the concomitant increase in the amount of thermal energy that had to be absorbed for deswelling. However, the valve closing time decreased with the increasing diameter of the PNM ball, possibly because of the increase in the rate of thermal energy dissipation for swelling of the PNM ball (Figure 3C). Given that PNM balls with diameters below 5.4 mm are fragile, difficult to return to as-printed sizes, and unstable for valve actuation, we selected a PNM ball diameter of 5.4 mm as optimal for valve actuation. The valve opening and closing times were invariant with the changes in the diameter of the flexible membrane (14–18 mm; Figure 3C). At fixed PNM ball (5.4 mm) and flexible membrane (14 mm) diameters, thick flexible membranes easily recovered upon deswelling of the PNM ball, which led to shorter valve opening times; however, the deformation of thick flexible membranes upon swelling of the PNM ball was difficult, which resulted in longer valve closing times (Figure 3D). As a result, the diameter of the flexible membrane was set to 14 mm (to decrease the volume of the flow chamber) and the membrane thickness was set to 50 μm.

At a constant PNM ball composition (50% NIPAM/4.0% MBA/2.0% graphene, w/v) and valve design (PNM ball diameter: 5.4 mm; flexible membrane diameter and thickness: 14 mm and 50 μm, respectively), the valve opening time did not change when the laser spot size increased from 1.0 to 5.0 mm at a fixed laser power of 2.5 W (Figure 3E). In contrast, a laser power increase from 1.0 to 5.0 W at the laser spot size of 1.0 mm resulted in shorter valve opening times and longer valve closing times because of the concomitant increase in the rate of thermal energy absorption for deswelling and passivation time required to release the absorbed thermal energy, respectively (Figure 3F). Consequently, the laser spot size and power were set at 1.0 mm and 2.5 W, respectively, to avoid the inhomogeneous heating of the PNM ball and actuate the four valves. Figure S6C shows that an increase in the loading flow rate from 0.25 to 1.5 mL min^–1^ did not change the valve closing and opening times, which indicated that the valve actuation conditions could be tailored to fit the metal-ion extraction conditions. When the loading flow rate exceeded 1.5 mL min^–1^, valve actuation was incomplete and unstable as the force generated by the stream passing through the flow chamber was close to or greater than that generated by the PNM ball pushing the flexible membrane to close the bottom channel. Thus, the maximal operation (sample loading and elution) flow rate was set at 1.0 mL min^–1^. The tunable valve response times allowed us to conduct an SPE scheme with individual steps performed at the desired times by adjusting the PNM ball composition, valve design, and laser actuation conditions.

Based on the arrangement of the four valves in the flow manifolds, the opening times of Valves 1, 2, and 3 determined the sample volume, evacuation volume, and elution volume, respectively. Considering the interstitial volume among the interlacing cuboids of the monolithic packing and volume of the channel connecting Valves 3 and 4 (0.67 mL; minimal elution volume), a time interval of 40 s (at an elution flow rate of 1.0 mL min^–1^) between the loading of the eluent through the monolithic packing and delivery of the eluted metal ions to the ICP-MS instrument was required. The following conditions based on the time intervals required for the SPE scheme were used; photocurable resins for printing the PNM ball: 50% NIPAM/4.0% MBA/2.0% graphene (w/v) for Valves 1 and 2 [opening time: 85 ± 3 s (sample and evacuation volumes), closing time: 232 ± 8 s] and 20% NIPAM/2.0% MBA/1.0% graphene (w/v) for Valves 3 and 4 [opening time: 245 ± 4 s (elution volume), closing time: 153 ± 5 s]. The sequential actuation of the four valves to match the time intervals of sample loading, evacuation, elution, and re-evacuation steps was used to realize a fully automatic SPE scheme by programming the NIR actuation of the four temperature-responsive switching valves without human intervention [Figures 2 and S3; time interval: 500 s; sample volume: 1.4 mL (opening time of Valve 1); evacuation volume: 1.4 mL (opening time of Valve 2); elution volume: 4.1 mL (opening time of Valve 3)]. Moreover, the sample and elution volumes were adjustable in the range of 1.4–5.8 mL through changing the opening times of Valves 1 and 4, which showcased the suitability of 4DP technologies for fabricating stimuli-responsive valves with tunable response times to match the time sequence for automation of an SPE scheme. These temperature-responsive switching valves were also actuated by battery-powered red laser pointers to demonstrate the applicability of our device in resource-limited settings or in the case of the unavailability of expensive valve apparatuses.

### Extraction of Metal Ions Using the 4D-Printed NIR-Actuated LOV-SPE Device

After optimizing the design, fabrication, and actuation conditions of the temperature-responsive switching valves, we optimized the monolithic packing to maximize the extraction efficiencies of the metal ions. The signal intensities of the metal ions increased with the increasing number of cuboids per layer (2 → 4 at 40 layers of interlacing cuboids) and cuboid packing layers (8 → 40 at 4 cuboids per layer) because of the increase in the surface area of the monolithic packing (Figure S7A,B). At a fixed cuboid length (9.1 mm) and height (0.6 mm) (4 cuboids per layer, 40 layers), the signal intensities of the metal ions increased when an increase in the cuboid width from 0.7 to 0.9 mm (Figure S7C) for the same reason but plateaued with an increase in the interstitial space between these cuboids from 1.1 to 1.4 mm, which suggested a negligible dispersion of eluting the metal ions from the cuboids with a wider interstitial space.^39,40,46^ Therefore, we fixed the monolithic packing at 4 cuboids [9.1 mm (length) × 0.9 mm (width) × 0.6 mm (height), interstitial space: 1.1 mm] per layer and 40 layers of these interlacing cuboids to maximize the extraction efficiencies of the metal ions.

For the sample loading step, Figure S7D reveals that extraction of the metal ions was tolerant of loading flow rates of up to 1.5 mL min^–1^ (i.e., >97% of the signal intensities remained when compared to the case of the sample loading flow rate of 0.1 mL min^–1^) because of the negligible flow resistance (back-pressure <1.0 psi) of the submillimeter-sized space between the interlacing cuboids. The loading flow rate was set at 1.0 mL min^–1^ to prolong the lifetime of the peristaltic tubing and minimize the tubing aging-induced flow rate drift. Figure S7E shows that the signal intensities of the metal ions extracted in the monolithic packing (fabricated using the Flex 57A resins) were roughly invariant (except for those of Cu) over a sample pH range of 2.0–10.0, possibly because the interactions between the cured copolymers (partially negatively charged C=O groups of acrylates; Figure S1B) and metal species [Mn^2+^, Co^2+^, Co(OH)_2_, Co(OH)_3_^–^, Ni^2+^, Ni(OH)_2_, Ni(OH)_3_^–^, Cu^2+^, CuOH^+^, Cu(OH)_2_, Cu(OH)_3_^–^, Zn^2+^, ZnOH^+^, Zn(OH)_2_, Zn(OH)_3_^–^, Cd^2+^, Cd(OH)_2_, Cd(OH)_3_^–^, Pb^2+^, PbOH^+^, Pb(OH)_2_, and Pb(OH)_3_^–^; modeled using Visual MINTEQ 3.1^61^] were unaltered by the hydronium and hydroxyl ions. We selected a sample acidity of pH 8.0 for the extraction of the metal ions to avoid the formation of hydroxide precipitates [e.g., Ca(OH)_2_, Mg(OH)_2_] during the analysis of real samples.

In the elution step, the extracted metal ions were eluted and transported to the ICP-MS system. When increasing elution flow rate, the signal intensities of the metal ions decreased because of the reduction in the detection time (Figure S7F).^46^Figure S7G,H reveal that an eluent of 0.5% HNO_3_ (v/v) and elution volume of >3.0 mL were needed to completely elute the extracted metal ions. At an elution flow rate of 1.0 mL min^–1^ and an eluent of 0.5% HNO_3_ (v/v), the signal intensities of the metal ions declined to 5% of the maximum values within 153 s (Figure S8A) without significant carry-over effects (Figure S8B). Therefore, an elution flow rate of 1.0 mL min^–1^, eluent of 0.5% HNO_3_ (v/v), and elution volume of 4.1 mL were selected to completely elute the metal ions from the monolithic packing (also considering the volume of the flow channel connecting Valves 3 and 4 and valve actuation conditions). Although the channels from the loading port to the monolithic packing contributed to the partial extraction of the metal ions (surface areas of the channels and monolithic packing: 0.2 and 1.6 cm^2^, respectively, according to Solidworks 2020) because the whole base was fabricated using the acrylate-based Flex 57A resins, it led to the insignificant carry-over effects when eluting the metal ions. To fabricate the base that provided the negligible extraction of the metal ions could be infeasible because most photocurable resins for vat photopolymerization 3DP were acrylate-based.^36,39,41,46,47^

Figure S7I,J indicate that the extraction of the metal ions was nearly unaltered by the dissolved salt matrix (>96% of the signal intensities of the metal ions were retained at salinities of up to 4.5% NaCl, w/v) and coexisting ions [K^+^ (1000 mg L^–1^), Ca^2+^ (1000 mg L^–1^), Mg^2+^ (1000 mg L^–1^), Fe^3+^ (100 mg L^–1^), Al^3+^ (100 mg L^–1^), HCO_3_^–^ (500 mg L^–1^), SO_4_^2–^ (2000 mg L^–1^), and Br^–^ (500 mg L^–1^); spiked recoveries: 96–105%], suggested that our device was applicable for the interference-free determination of the metal ions in high-salt-content samples. Through loading of the samples into the monolithic packing to acquire the sample volume resulting in the saturation of the signal intensities of the metal ions, the adsorption capacities (corrected by the extraction efficiencies) of the monolithic packing were obtained as 139.5 μg of Mn cm^–2^, 144.9 μg of Co cm^–2^, 77.8 μg of Ni cm^–2^, 96.8 μg of Zn cm^–2^, 63.1 μg of Cu cm^–2^, 100.3 μg of Cd cm^–2^, and 118.2 μg of Pb cm^–2^, corresponding to the extraction of the metal ions from a 1.0 mL sample with concentrations from 101 mg L^–1^ (Cu) to 232 mg L^–1^ (Co) (surface area of the monolithic packing: 1.6 cm^2^).

**Figure 4:**
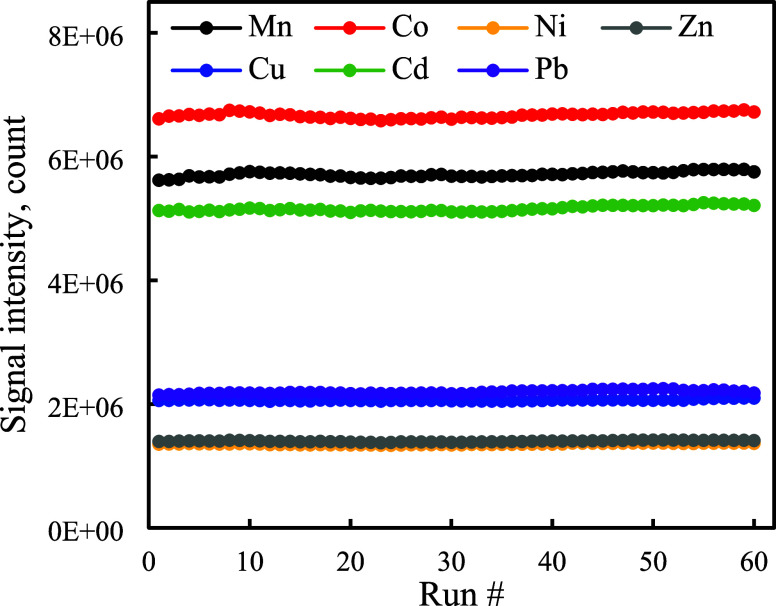
Signal intensities of the metal ions (10 μg L^–1^) obtained from the 4D-printed NIR-actuated LOV-SPE device over 60 continuous cycles (8.3 h).

The relative standard deviations (RSDs) of the signal intensities of the metal ions determined by eight devices were less than 6.6% (interdevice variations), indicating that the print-pause-print DLP 3DP technique with the photocurable resins was suitable for the fabrication of the NIR-actuated temperature-responsive valves and monolithic packing. The RSDs of the signal intensities of the metal ions measured from 60 continuous cycles were below 1.3% (Figure 4; intradevice variations), indicating the good reversible stimuli-responsive properties of the PNM ball and device reusability. The interday fluctuations (RSDs) of the slopes of the calibration curves of the metal ions were less than 11.1% over 57 days [Figure S8C; including the construction of the calibration curves, analyses of the reference materials and real samples (with their spike analyses)], demonstrating the negligible fouling of the monolithic packing and flow manifolds by real sample matrices and the durability of the SPE device and its suitability for long-term operation. The combined results suggested that the consistent, reusable, and durable 4D-printed NIR-actuated LOV-SPE devices were well suited for the extraction of the metal ions for ICP-MS analysis.

### Analytical Characteristics

The analytical performance of our analytical method was evaluated under the above-mentioned optimized conditions (Table S3). Table 1 shows that the linear correlation coefficients (*R*_s_) of the metal ions were all greater than 0.9998 within the working ranges (Figure S9A,B). The method detection limits (MDLs; 3 × standard deviation of baseline noise from seven blank measurements), extraction efficiencies (ratios between the elution peak areas of the metal ions with and without extraction; 10 μg L^–1^), and enhancement factors (EFs; ratios of the elution peak heights of the metal ions after and before extraction; 10 μg L^–1^) were 0.1–6.8 ng L^–1^, 92.7–94.8%, and 7.1–10.5, respectively. Tables 1 and S4 show that the measured concentrations of all metal ions in the four reference materials agreed with the certified values [relative error: −2.2 to +3.3%; all *p* values: >0.5164 (statistical significance level: *p* < 0.05)]. Moreover, Tables 1 and S5 reveal that the spike recoveries of environmental water and human urine samples ranged from 97 to 103%. Thus, our method was concluded to tolerate real sample matrices for the highly sensitive and reliable determination of the metal ions in environmental water and human urine samples. The *R*_s_ (>0.9999) and MDLs (0.3–6.8 ng L^–1^) obtained from the commercial automatic LOV-SPE system were similar to those obtained from the 4D-printed LOV-SPE device (Tables S6, S7, and Figure S9C,D), suggesting the competitiveness of the latter. Furthermore, compared with previously reported automatic LOV-SPE systems with 3D-printed SPE devices^36−47^ or commercial SPE columns,^62,63^ our method offered the advantages of lower MDLs, higher adsorption capacities, higher extraction efficiencies, and higher throughput (Table S8). Thus, the 4D-printed NIR-actuated LOV-SPE device was concluded to be a viable alternative to commercial LOV-SPE systems for the sensitive and reliable determination of metal ions in high-salt-content real samples, allowing one to considerably reduce the cost and complexity of developing an automatic LOV-SPE scheme.

**Table 1:** Analytical Characteristics of the SPE Schemes Performed Using the 4D-Printed NIR-Actuated LOV-SPE Device

element	working range (ng L^–1^)	calibration curve	*R*	MDL (ng L^–1^)	extraction efficiency (%)	EF	relative error (%)^b^	RSD (%)^c^	spike recovery (%)^d^
^55^Mn	50–5000	*y* = 571[Mn]^a^ + 8485	1.0000	1.5	94.5	7.4	–0.8 to +1.8	4.3	97–103
^59^Co	1–100	*y* = 668[Co]^a^ + 593	1.0000	0.2	93.2	8.1	+0.1 to +2.9	4.8	99–102
^60^Ni	50–5000	*y* = 136[Ni]^a^ + 2555	1.0000	2.7	93.6	8.9	–0.7 to +1.3	2.6	100–103
^64^Zn	50–5000	*y* = 140[Zn]^a^ + 7410	1.0000	6.8	92.5	10.1	–0.5 to +1.4	3.7	101–103
^65^Cu	50–5000	*y* = 206[Cu]^a^ + 4804	1.0000	2.9	92.7	9.4	–0.3 to +2.0	3.2	97–101
^114^Cd	1–100	*y* = 516[Cd]^a^ + 217	0.9999	0.1	94.8	10.5	–2.2 to +1.0	3.1	97–99
^208^Pb	1–100	*y* = 217[Pb]^a^ + 1330	0.9999	0.9	92.9	7.1	+0.2 to +3.3	4.4	98–101

a ng L^–1^.

b Obtained from comparing the measured concentrations with the certified values from the four reference materials.

c The maximal value from the measured concentrations of the four reference materials.

d Obtained from the collected environmental water and human urine samples (spiked concentrations: 0.05 μg L^–1^ for Co, Cd, and Pb and 0.5 μg L^–1^ for Mn, Ni, Cu, and Zn).

We used 4DP technologies to overcome the problem of needing expensive commercial valve apparatuses with their control systems and the skills of arranging and programming flow control valves in flow manifolds for constructing automatic LOV-SPE systems. Compared with those reported 3D-printed SPE devices and our previous studies,^26,36−47^ the major achievement and novelty of this study were the first to demonstrate the possibility of using 4DP technologies and stimuli-responsive materials to directly fabricate an NIR-actuated LOV-SPE device to enable the automation of an SPE-based sample pretreatment scheme for trace metal analysis without using any commercial valve apparatuses, showing the capability and applicability of 4DP technologies to revolutionize the development of sample pretreatment devices and related analytical schemes. Furthermore, this study revealed the overlapping area of the 4DP technologies, stimuli-responsive flow control valves, and FIA-LOV techniques to open new possibilities and applications of automatic FIA-LOV systems, which had significant implications for boosting the development of new-generation automatic analytical devices for modern analytical sciences. To enable more applications, this 4DP-based FIA-LOV device is potentially applicable to be a key component for the automation of most batchwise labor-intensive analytical works, for example, to replace the monolithic packing with an enzyme-immobilized bioreactor for the automation of an online enzymatic derivatization and sensing scheme for determining specific targets and with antigen-coated channels or wells for the automation of an enzyme-linked immunosorbent assay for detecting and quantifying certain antibodies or antigens.

## Conclusions

A fully 4D-printed NIR-actuated LOV-SPE device was developed to realize a fully automatic SPE scheme coupled with ICP-MS for the sensitive, interference-free, and reliable determination of the metal ions in high-salt-content real samples, showing the following advantages over automatic LOV-SPE systems equipped with commercial valves or valve devices fabricated by traditional methods. (i) Our SPE scheme was fully automatized by programming the NIR actuation of the temperature-responsive valves and did not require commercial valves and control systems, which substantially reduced the costs and complexity of developing SPE schemes for the subsequent trace metal analysis. (ii) The remote control of the NIR actuation of the temperature-responsive valves was simple and reversible, and the valve response times could be tuned to fit the time interval required for each SPE step. (iii) Our device exhibited good reusability, durability, and analytical performance, thus being a viable and smaller (around one-15th in the size) alternative to commercial automatic LOV-SPE systems and enabling sensitive and reliable trace metal analysis. (iv) All parts of our device were fabricated using a low-cost DLP 3D printer and were ready for assembly and actuation, illustrating the suitability of 4DP technologies for developing stimuli-responsive devices and miniaturization for diverse analytical applications. Most importantly, this work realized the 4D-printed FIA-LOV device to enable a fully automatic SPE scheme through programming the NIR actuation of temperature-responsive valves without using commercial valve apparatuses. We believe that the presented paradigm can practically extend the functionality of conventional 3D-printed devices to meet future requirements and applications of LOV-based analytical systems.

## Supporting Information Available

Infrared spectra of these cured resins; thermal analysis of the cured NIPAM/MBA photocurable resins; detailed dimensions, time sequence, operating sequence, optimized operating conditions and elution profiles, temporal responses, and daily calibration slopes of the metal ions for the 4D-printed NIR-actuated LOV-SPE device; schematic representations, operating sequence, operating conditions, and analytical characteristics for the commercial automatic LOV-SPE system; measured valve response time plotted with respect to the contents of NIPAM and MBA (with 0.5% graphene), graphene concentration, and sample loading flow rate; relative signal intensities of the metal ions plotted with respect to the number of cuboids per layer, number of layers of interlacing cuboids, width of cuboids and interstitial space between cuboids, sample loading flow rate, sample pH, elution flow rate, concentration of HNO_3_ in the eluent, elution volume, concentration of NaCl, and interference ions; calibration plots; analytical results of four reference materials and environmental water and human urine samples; analytical characteristics of the commercial automatic LOV-SPE systems with 3D-printed SPE devices and commercial SPE columns for trace metal analysis (PDF)
