# Supplementary material for: Fully 4D-Printed Near-Infrared-Actuated Lab-on-Valve Solid-Phase Extraction Devices
Source: Anal Chem. 2024 Dec 25;97(2):1281–90. doi: 10.1021/acs.analchem.4c05363 (PMC11755401; doi:10.1021/acs.analchem.4c05363)
Supplement: Supplementary file 1 — ac4c05363_si_001.pdf [file ac4c05363_si_001.pdf]

Supporting Information

Fully 4D-Printed Near-Infrared-Actuated Lab-on-Valve Solid-Phase Extraction Devices

Chia-Hsun Kuo and Cheng-Kuan Su*

Department of Chemistry, National Chung Hsing University, Taichung City 402202, Taiwan, R.O.C.

Corresponding Author

Cheng-Kuan Su

E-mail: cksu@nchu.edu.tw

Table of Content

Page S-4	**Figure S1.** Infrared spectra of the cured (A) graphene-incorporated NIPAM/MBA resins, (B) Flex 57A resins, and (C) Aqua Clear resins, recorded using a Fourier transform infrared spectrometer (Tensor 27, Bruker).
Page S-5	**Figure S2.** Detailed device dimensions, including the (A) base (top view), (B) base (front view), (C) valve cover with the PNM ball, (D) monolithic packing, and (E) cross-sectional view of the monolithic packing.
Page S-6	**Figure S3.** Time sequence of the SPE scheme performed using the 4D-printed NIR-actuated LOV-SPE device through programming the NIR actuation of the four temperature-responsive switching valves.
Page S-7	**Figure S4.** Schematic representations of the commercial automatic LOV-SPE system comprising a 3D-printed SPE column and three electric switching valves.
Page S-9	**Figure S5.** Thermal analysis (differential scanning calorimetry) of the cured NIPAM/MBA photocurable resins without and with incorporation of 1.0% graphene (w/v), recorded using a thermal analyzer (HT-2, Mettler Toledo).
Page S-10	**Figure S6.** Measured valve response time plotted with respect to the (A) contents of NIPAM and MBA (with 0.5% graphene, w/v), (B) graphene concentration (fixed at 50% NIPAM/4.0% MBA, w/v), and (C) sample loading flow rate.
Page S-11	**Figure S7.** Relative signal intensities of the metal ions plotted with respect to the (A) number of cuboids per layer, (B) number of layers of interlacing cuboids, (C) width of cuboids and interstitial space between cuboids, (D) sample loading flow rate, (E)

	sample pH, (F) elution flow rate, (G) concentration of HNO_3 in the eluent, (H) elution volume, (I) concentration of NaCl, and (J) interference ions.
Page S-13	**Figure S8.** (A) Elution profiles and (B) temporal responses of the metal ions (10 μg L^{-1}) obtained using the 4D-printed NIR-actuated LOV-SPE device. (C) Daily calibration slopes of the metal ions from the same 4D-printed NIR-actuated LOV-SPE device used for up to 57 days.
Page S-14	**Figure S9.** (A), (C) Calibration plots of Mn, Ni, Zn, and Cu (50–5000 ng L^{-1}) and (B), (D) calibration plots of Co, Cd, and Pb (1–100 ng L^{-1}).
Page S-15	**Table S1.** Operating sequence of the 4D-printed NIR-actuated LOV-SPE device
Page S-16	**Table S2.** Operating sequence of the commercial automatic LOV-SPE system
Page S-17	**Table S3.** Optimized operating conditions for the 4D-printed NIR-actuated LOV-SPE device
Page S-19	**Table S4.** Analytical results of four reference materials (n = 5)
Page S-21	**Table S5.** Analytical results of environmental water and human urine samples (n = 5)
Page S-22	**Table S6.** Operating conditions for the commercial automatic LOV-SPE system
Page S-24	**Table S7.** Analytical characteristics of the commercial automatic LOV-SPE system
Page S-25	**Table S8.** Analytical characteristics of the automatic LOV-SPE systems comprising commercial valves with 3D-printed SPE devices and commercial SPE columns for trace metal analysis

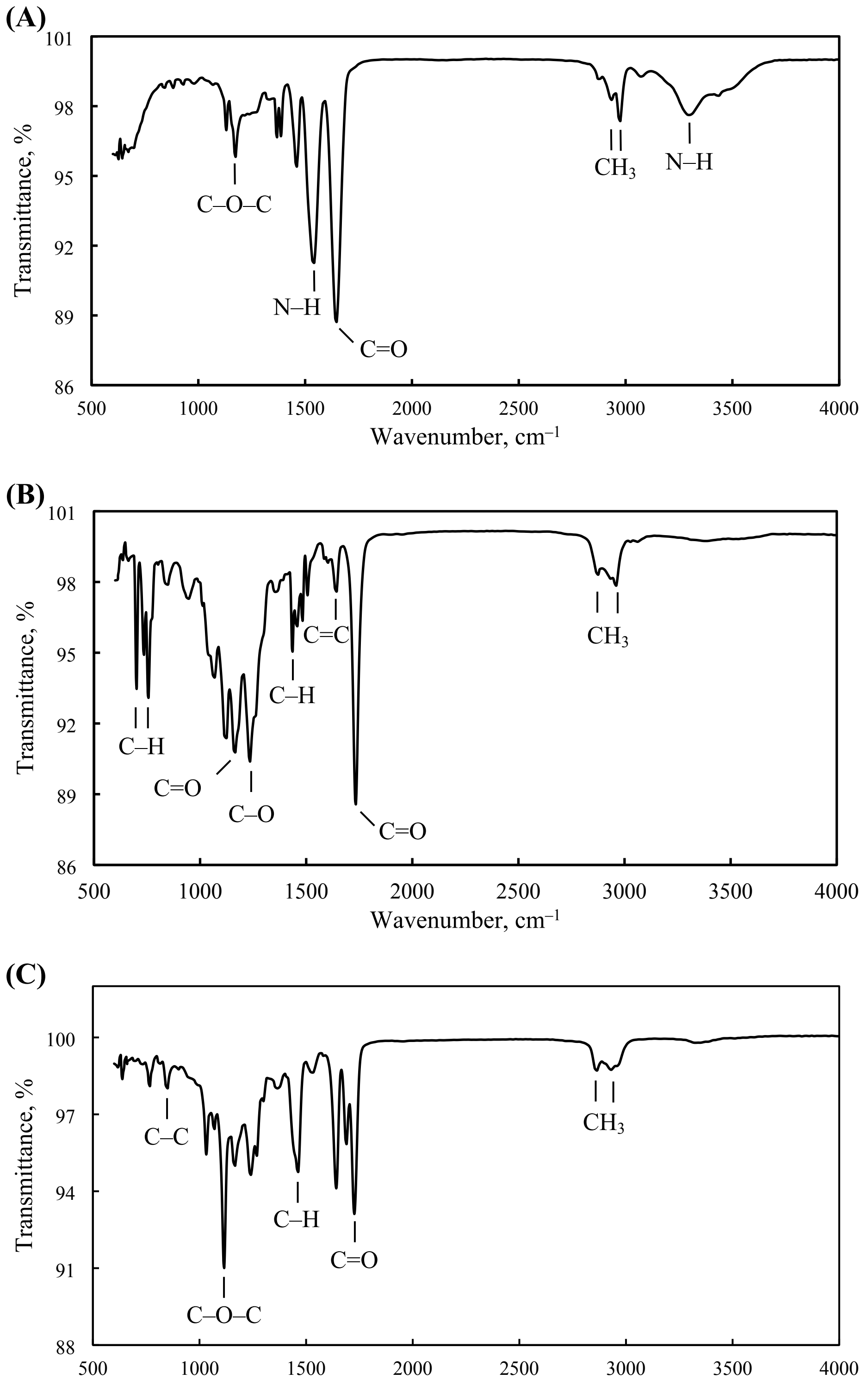

Figure S1. Infrared spectra of the cured (A) graphene-incorporated NIPAM/MBA resins, (B) Flex 57A resins, and (C) Aqua Clear resins, recorded using a Fourier transform infrared spectrometer (Tensor 27, Bruker).

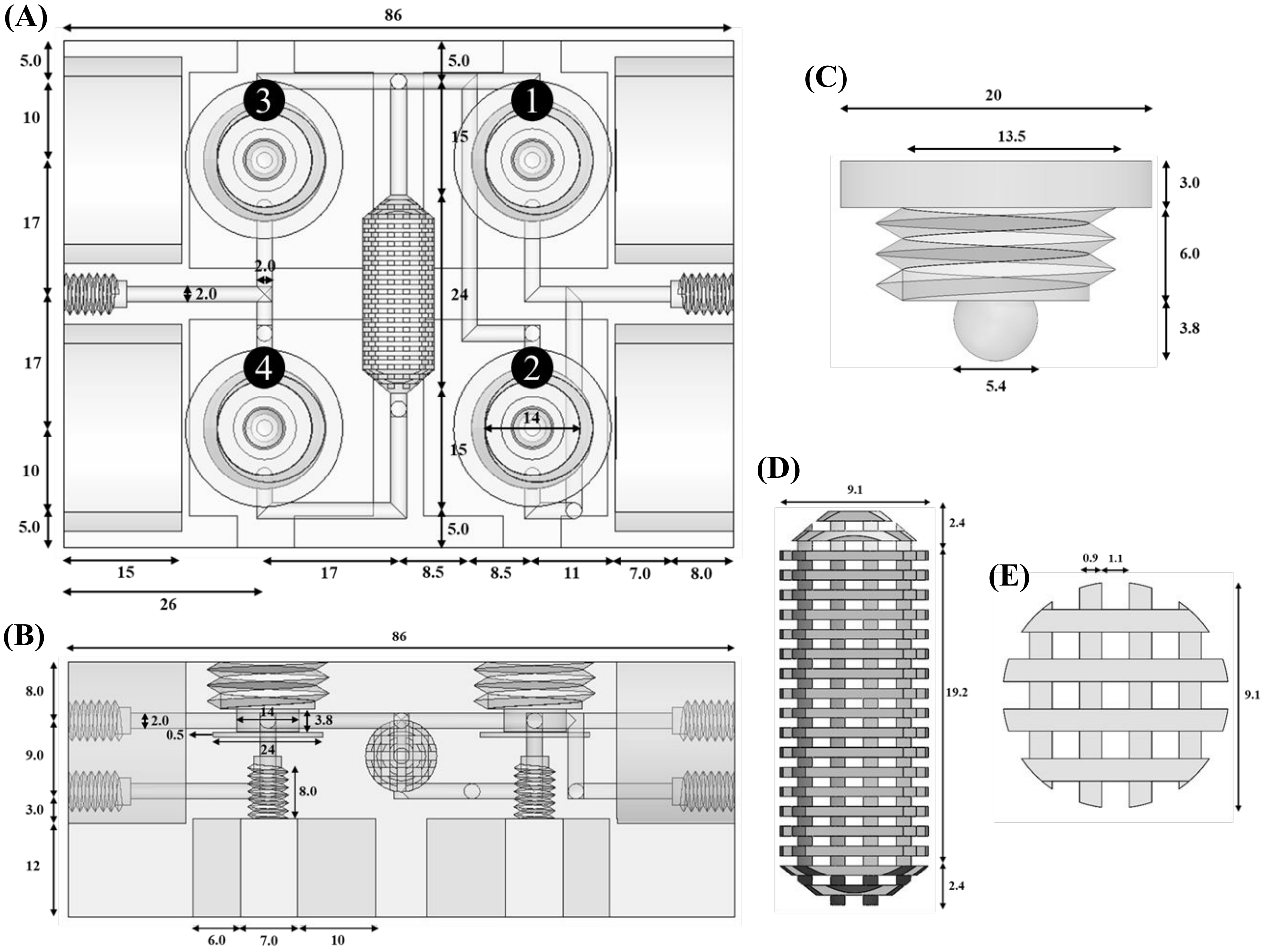

Figure S2. Detailed device dimensions, including the (A) base (top view), (B) base (front view), (C) valve cover with the PNM ball, (D) monolithic packing, and (E) cross-sectional view of the monolithic packing. Units: mm.

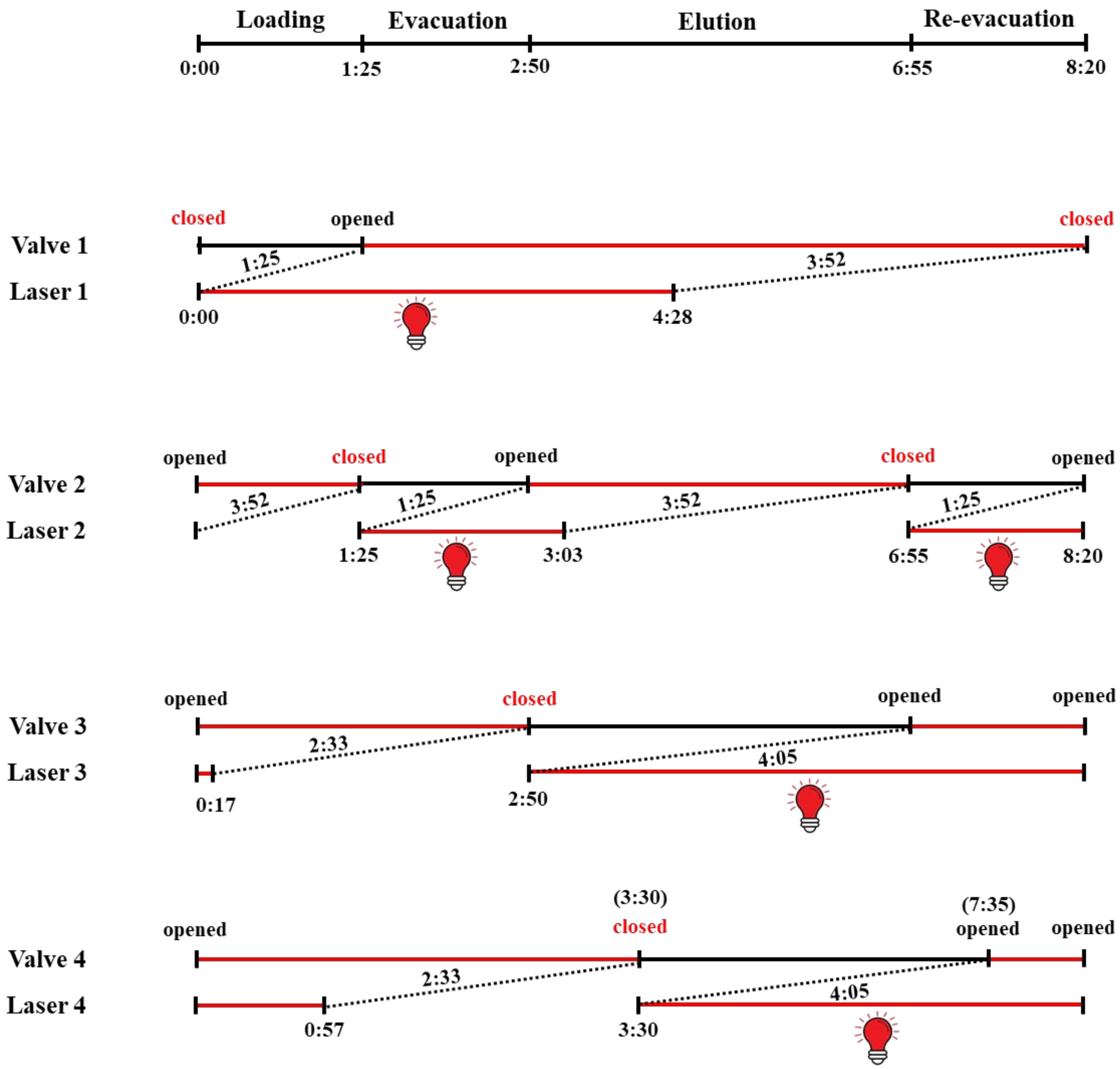

Figure S3. Time sequence of the SPE scheme performed using the 4D-printed NIR-actuated LOV-SPE device through programming the NIR actuation of the four temperature-responsive switching valves.

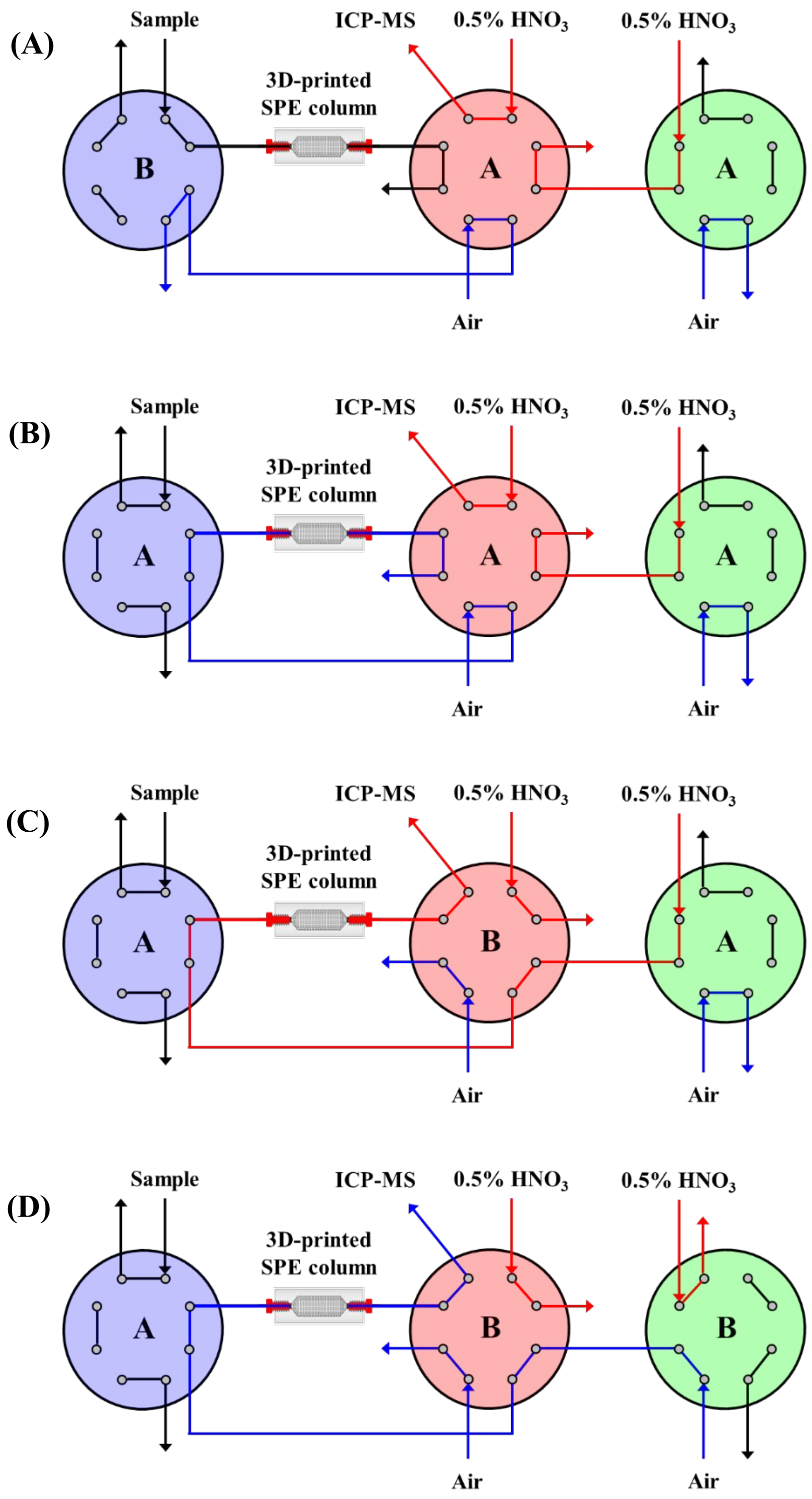

Figure S4. Schematic representations of the commercial automatic LOV-SPE system comprising a 3D-printed SPE column and three electric switching valves. (A) The conditioned sample (pH

8.0) was loaded (1.0 mL min^{-1}) into the 3D-printed SPE column for extraction of the metal ions. (B) Residual sample matrices were evacuated (1.0 mL min^{-1}) using an air stream. (C) The extracted metal ions were eluted (1.0 mL min^{-1}) using 0.5% HNO_3 (v/v) and directly delivered into the ICP-MS system. (D) The residual eluent in the 3D-printed SPE column was replaced by an air stream (1.0 mL min^{-1}) for loading of the next sample. V1, V2, and V3: two-position, eight-port electric switching valves; unmarked arrow: outflow of liquid waste.

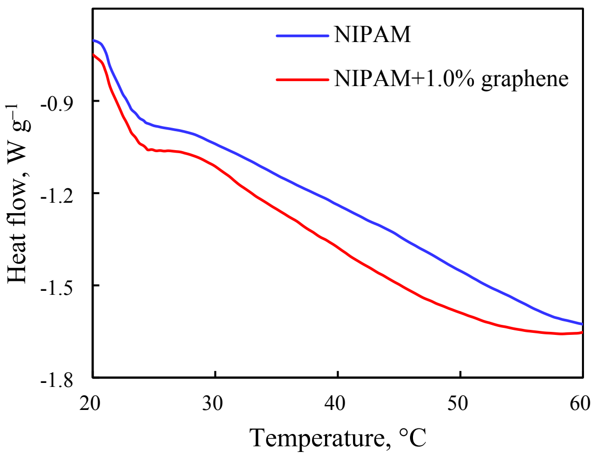

Figure S5. Thermal analysis (differential scanning calorimetry) of the cured NIPAM/MBA photocurable resins without and with incorporation of 1.0% graphene (w/v), recorded using a thermal analyzer (HT-2, Mettler Toledo).

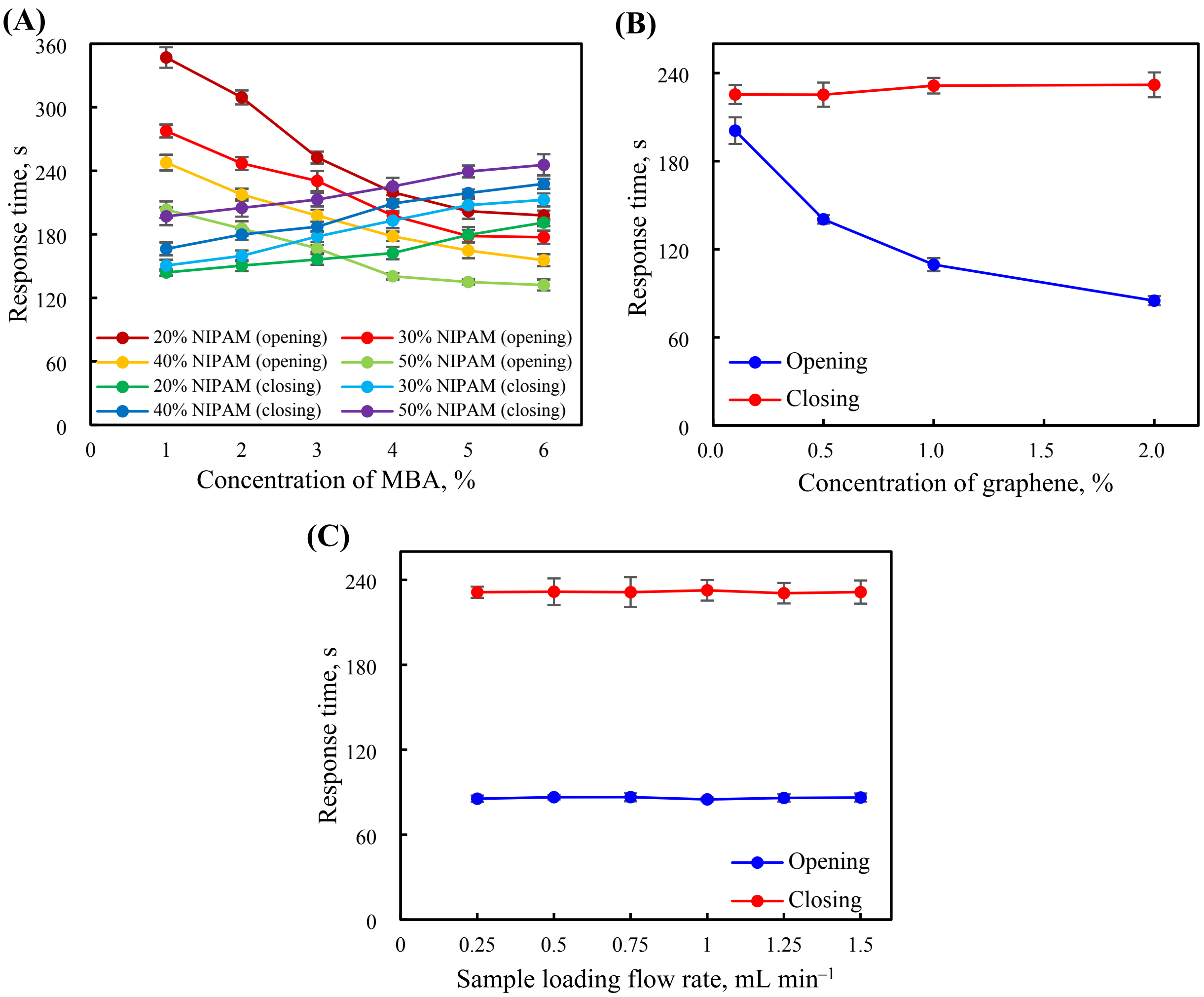

Figure S6. Measured valve response time plotted with respect to the (A) contents of NIPAM and MBA (with 0.5% graphene, w/v), (B) graphene concentration (fixed at 50% NIPAM/4.0% MBA, w/v), and (C) sample loading flow rate. The valve response time was measured as the time interval required for switching the flow from horizontal to vertical direction (valve opening) after NIR irradiation or switching the flow from vertical to horizontal direction (valve closing) after terminating the NIR source. Error bars represent standard deviations (n = 8).

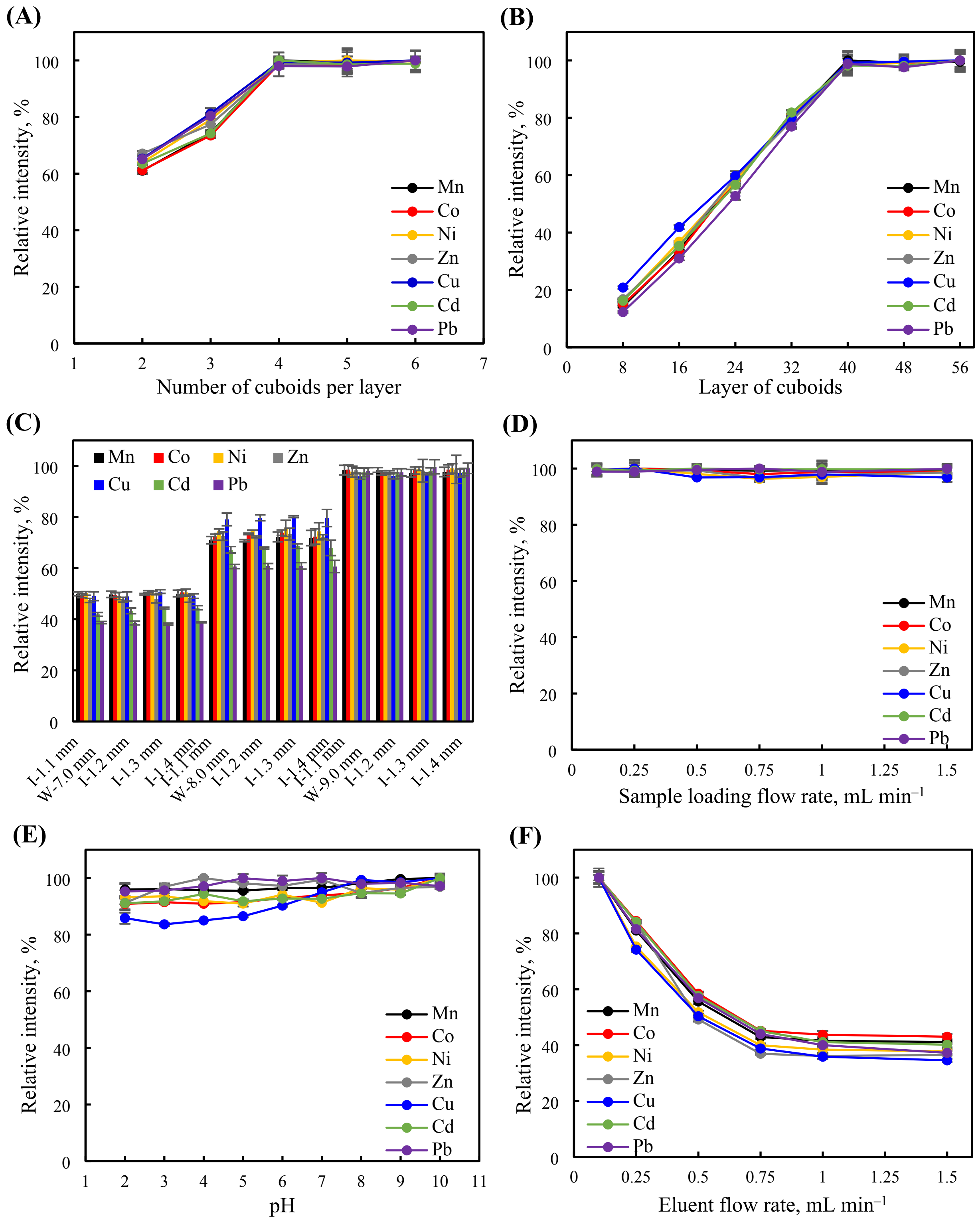
(A)
Relative intensity, %
Number of cuboids per layer
0
20
40
60
80
100
1
2
3
4
5
6
7
Mn
Co
Ni
Zn
Cu
Cd
Pb
(B)
Relative intensity, %
Layer of cuboids
0
20
40
60
80
100
0
8
16
24
32
40
48
56
Mn
Co
Ni
Zn
Cu
Cd
Pb
(C)
Relative intensity, %
0
20
40
60
80
100
Mn
Co
Ni
Zn
Cu
Cd
Pb
I-1.1 mm
W-7.0 mm
I-1.2 mm
I-1.3 mm
I-1.4 mm
I-1.1 mm
W-8.0 mm
I-1.2 mm
I-1.3 mm
I-1.4 mm
I-1.1 mm
W-9.0 mm
I-1.2 mm
I-1.3 mm
I-1.4 mm
(D)
Relative intensity, %
Sample loading flow rate, mL min−1
0
20
40
60
80
100
0
0.25
0.5
0.75
1
1.25
1.5
Mn
Co
Ni
Zn
Cu
Cd
Pb
(E)
Relative intensity, %
pH
0
20
40
60
80
100
1
2
3
4
5
6
7
8
9
10
11
Mn
Co
Ni
Zn
Cu
Cd
Pb
(F)
Relative intensity, %
Eluent flow rate, mL min−1
0
20
40
60
80
100
0
0.25
0.5
0.75
1
1.25
1.5
Mn
Co
Ni
Zn
Cu
Cd
Pb

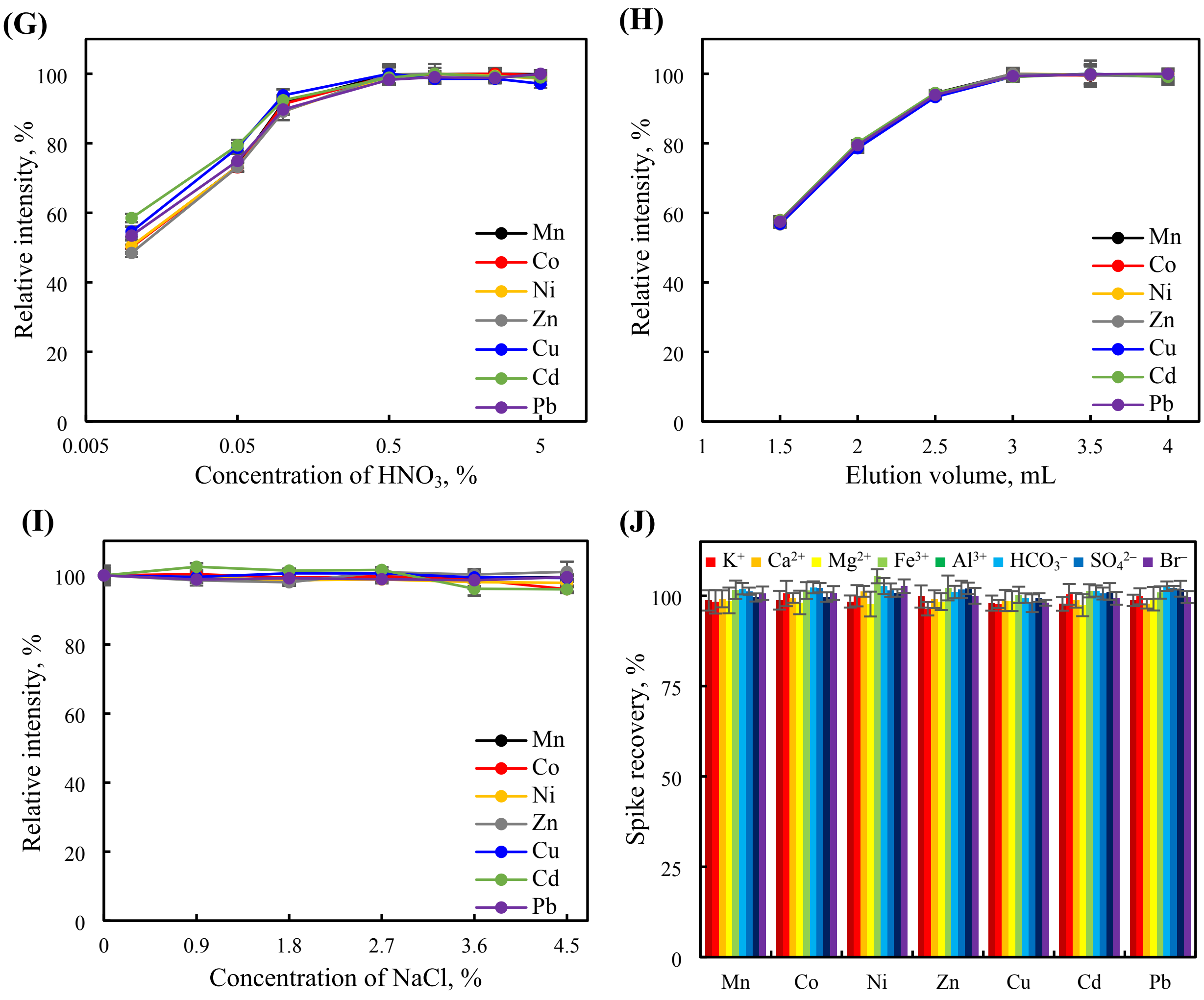

Figure S7. Relative signal intensities of the metal ions plotted with respect to the (A) number of cuboids per layer, (B) number of layers of interlacing cuboids, (C) width of cuboids and interstitial space between cuboids, (D) sample loading flow rate, (E) sample pH, (F) elution flow rate, (G) concentration of HNO_3 in the eluent, (H) elution volume, (I) concentration of NaCl, and (J) interference ions. Data in (A)-(I) have been normalized to the respective maxima of the metal ions (10 μg L^{-1}) for each parameter. Error bars represent standard deviations (n = 5).

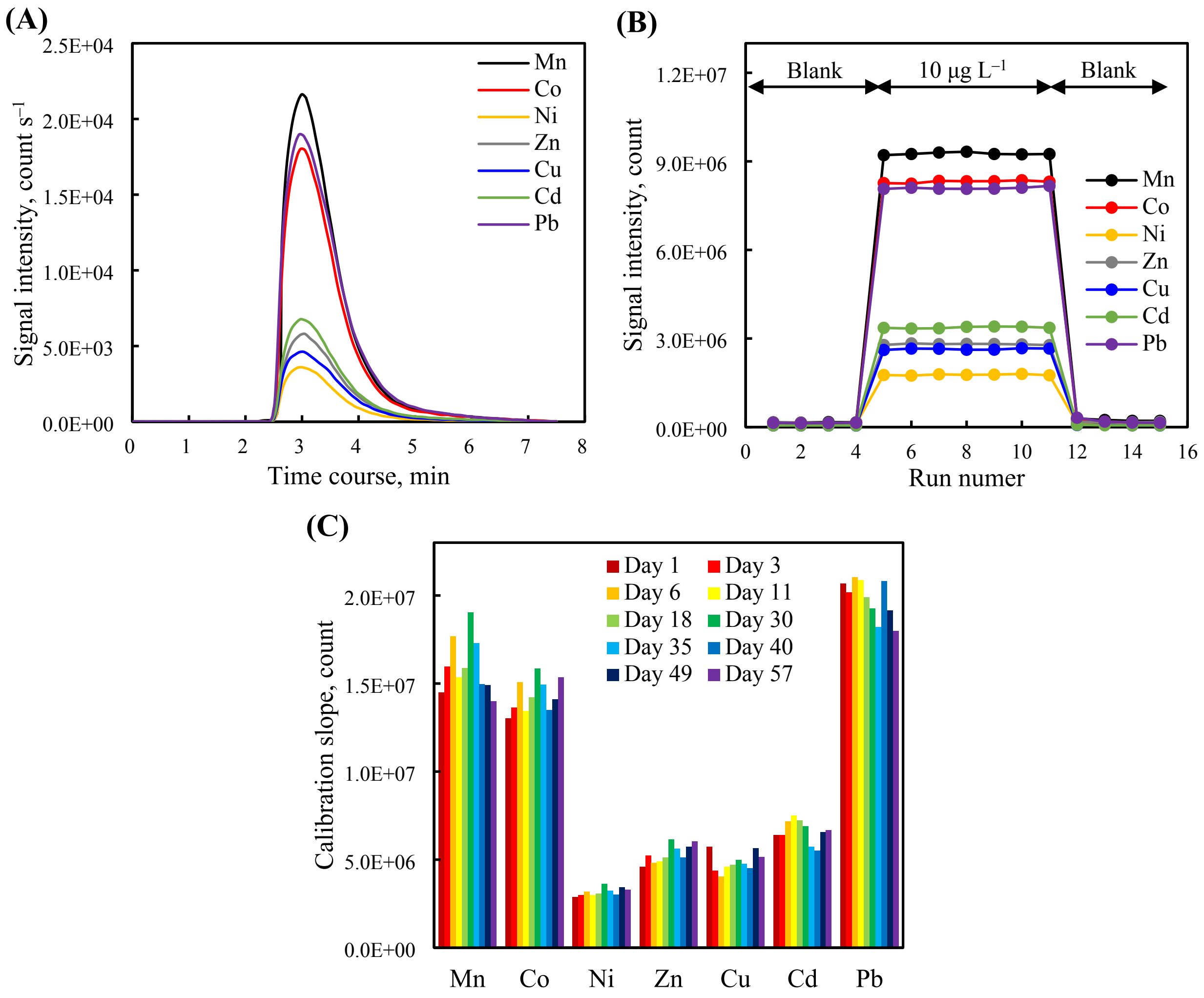

Figure S8. (A) Elution profiles and (B) temporal responses of the metal ions (10 μg L^{-1}) obtained using the 4D-printed NIR-actuated LOV-SPE device. (C) Daily calibration slopes of the metal ions from the same 4D-printed NIR-actuated LOV-SPE device used for up to 57 days. The fluctuations (RSDs) of the calibration slopes for the metal ions were 10.0% for Mn, 6.6% for Co, 7.3% for Ni, 9.9% for Zn, 11.1% for Cu, 9.6% for Cd, and 5.6% for Pb.

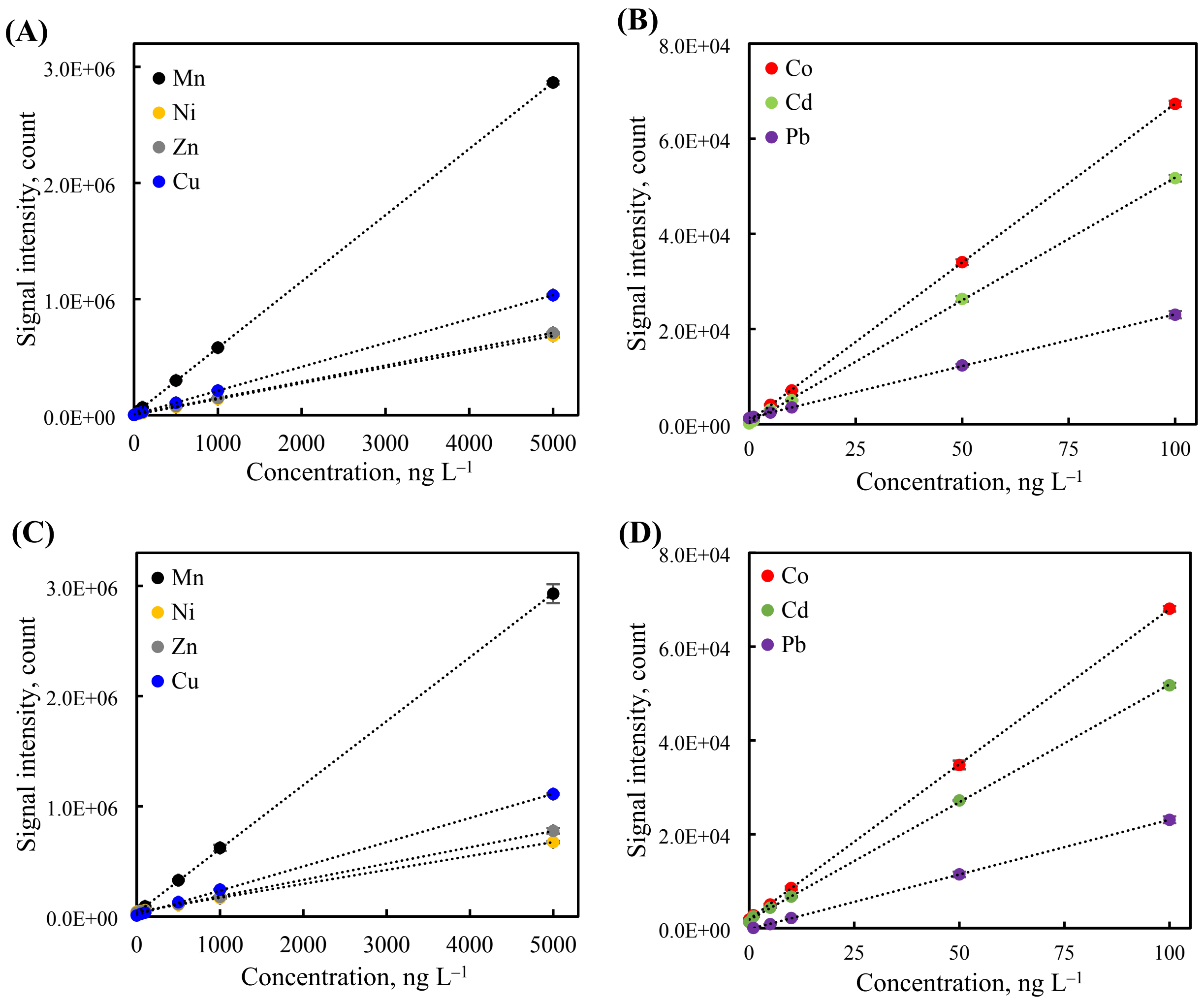

Figure S9. (A), (C) Calibration plots of Mn, Ni, Zn, and Cu (50–5000 ng L^{-1}) and (B), (D) calibration plots of Co, Cd, and Pb (1–100 ng L^{-1}). (A) and (B) were obtained from the 4D-printed NIR-actuated LOV-SPE device; (C) and (D) were obtained from the commercial automatic LOV-SPE system. Error bars represent standard deviations (n = 5).

Table S1. Operating sequence of the 4D-printed NIR-actuated LOV-SPE device

Step	Time interval	Valve 1	Valve 2	Valve 3	Valve 4	Functions
sample loading (Figure 2A)	0:00–1:25	opened	closed	closed	closed	loading of the conditioned sample for extraction of the metal ions through the loading port of Valve 1 and removal of sample matrices through the waste port of Valve 4
evacuation (Figure 2B)	1:25–2:50	closed	opened	closed	closed	loading of an air stream through the loading port of Valve 2 to remove sample matrices through the waste port of Valve 4
elution (Figure 2C)	2:50–6:55	closed	closed	opened	opened	loading of the eluent through the loading port of Valve 3 to elute the metal ions and transportation the eluted metal ions into the ICP-MS system through the inlet port of Valve 4
re-evacuation (Figure 2D)	6:55–8:20	closed	opened	closed	closed	loading of an air stream through the loading port of Valve 2 to evacuate the residual eluent for loading of the next sample

Table S2. Operating sequence of the commercial automatic LOV-SPE system

Step	Valve position (1 → 3)	Time interval	Function
sample loading (Figure S4A)	BAA	0:00–1:25	loading of the conditioned sample into the SPE column
evacuation (Figure S4B)	AAA	1:25–2:50	evacuating the SPE column with an air stream to remove sample matrices
elution (Figure S4C)	ABA	2:50–6:55	eluting the metal ions and delivering them into the ICP-MS
re-evacuation (Figure S4D)	ABB	6:55–8:20	replacing the residual eluent with an air stream for next loading

Table S3. Optimized operating conditions for the 4D-printed NIR-actuated LOV-SPE device

NIR-actuated temperature-responsive switching valve	
Concentration of NIPAM/MBA/graphene (Valve 1 & 2)	50%/4.0%/2.0% (w/v)
Concentration of NIPAM/MBA/graphene (Valve 3 & 4)	20%/2.0%/1.0% (w/v)
VPTT of PNM ball	27.7 °C
Distance from cover end to flexible membrane	3.8 mm
Diameter of PNM ball	5.4 mm (1.6 mm inserted in the valve cover)
Diameter and thickness of flexible membrane	14 mm/50 μm
Laser spot size and power	1.0 mm/2.5 W

Monolithic packing	
Dimension of interlacing cuboids	9.1 mm (L) × 0.9 mm (W) × 0.6 mm (H)
Interstitial distance between cuboids	1.1 mm
Number of cuboids per layer	4 cuboids
Number of layers of interlacing cuboids	40 layers

SPE scheme	
Sample condition	pH 8.0
Conditioning buffer	10 mM phosphate buffer
Sample loading flow rate and volume	1.0 mL min^{-1}, 1.4 mL
Evacuation medium	Air
Evacuation flow rate and volume	1.0 mL min^{-1}, 1.4 mL
Eluent	0.5% HNO_3 (v/v)
Elution flow rate and volume	1.0 mL min^{-1}, 4.1 mL
Re-evacuation medium	Air
Re-evacuation flow rate and volume	1.0 mL min^{-1}, 1.4 mL

ICP-MS	
ICP mass spectrometer	Agilent 7700x
Plasma forward power	1500 W
Plasma gas flow rate	15 L min^{-1}
Auxiliary gas flow rate	0.9 L min^{-1}
Carrier gas flow rate	0.92 L min^{-1}
Makeup gas flow rate	0.18 L min^{-1}
Sampling cone	Pt, 1-mm orifice
Skimmer cone	Pt, 0.4-mm orifice
Analysis mode	Time-resolved analysis
Integration time	50 ms
Isotopes monitored	^{55}Mn, ^{59}Co, ^{60}Ni, ^{64}Zn, ^{65}Cu, ^{114}Cd, ^{208}Pb

Table S4. Analytical results of four reference materials (n = 5)

		CASS-6	SLRS-5	1643f	Trace Elements Urine L-2
Mn	Certified value, μg L^{-1}	2.22 ± 0.12	0.081 ± 0.006	37.14 ± 0.60	10.9 ± 2.2
	Measured value, μg L^{-1}	2.25 ± 0.02	$0.08 \pm 0.00_4$	37.22 ± 0.72	10.81 ± 0.12
	Relative error, %	+1.5	+1.8	+0.2	–0.8
	p value	0.5964	0.7644	0.8534	0.9295
Co	Certified value, μg L^{-1}	0.0672 ± 0.0052	0.05[a]	25.30 ± 0.17	10.6 ± 2.1
	Measured value, μg L^{-1}	$0.07 \pm 0.00_2$	$0.05 \pm 0.00_2$	25.32 ± 0.72	10.62 ± 0.22
	Relative error, %	+2.1	+2.9	+0.1	+0.1
	p value	0.5896	--	0.9533	0.9836
Ni	Certified value, μg L^{-1}	0.418 ± 0.040	0.476 ± 0.064	59.8 ± 1.4	41.3 ± 8.3
	Measured value, μg L^{-1}	0.42 ± 0.01	0.47 ± 0.01	58.50 ± 1.57	41.85 ± 0.59
	Relative error, %	+1.2	–0.7	–0.5	+1.3
	p value	0.7931	0.9197	0.7580	0.8862
Zn	Certified value, μg L^{-1}	1.27 ± 0.18	0.845 ± 0.095	74.4 ± 1.7	1338 ± 269
	Measured value, μg L^{-1}	1.29 ± 0.03	0.84 ± 0.03	74.58 ± 1.37	1340.00 ± 2.00
	Relative error, %	+1.4	–0.5	+0.2	+0.1
	p value	0.8126	0.9309	0.8583	0.9871
Cu	Certified value, μg L^{-1}	0.530 ± 0.032	17.4 ± 1.3	21.66 ± 0.71	22[a]
	Measured value, μg L^{-1}	0.54 ± 0.02	17.67 ± 0.36	21.59 ± 0.22	21.19 ± 0.54
	Relative error, %	+2.0	+1.6	–0.3	+0.9
	p value	0.5164	0.6663	0.8385	--
Cd	Certified value, μg L^{-1}	0.0217 ± 0.0018	0.0060 ± 0.0014	5.89 ± 0.13	4.9 ± 0.2
	Measured value, μg L^{-1}	$0.02 \pm 0.00_1$	$0.01 \pm 0.00_{02}$	5.93 ± 0.04	4.89 ± 0.15
	Relative error, %	+1.0	–2.2	+0.6	+0.1

	p value	0.8196	0.8783	0.5293	0.9309
Pb	Certified value, μg L^{-1}	0.0106 ± 0.0040	0.081 ± 0.006	18.488 ± 0.084	90.7 ± 18.3
	Measured value, μg L^{-1}	0.01 ± 0.00_{04}	0.08 ± 0.00_{4}	18.55 ± 0.54	90.84 ± 0.85
	Relative error, %	+3.3	+2.7	+0.3	+0.2
	p value	0.8716	0.5524	0.8061	0.9869

^{a}: Reference value

Table S5. Analytical results of environmental water and human urine samples (n = 5)

		Seawater	River water	Ground water	Municipal wastewater	Urine
Mn	Measured conc., μg L^{-1}	0.36 ± 0.01	0.25 ± 0.01	$0.08 \pm 0.00_2$	1.39 ± 0.03	0.34 ± 0.01
	Conc. after spiking, μg L^{-1}	0.86 ± 0.03	0.74 ± 0.01	0.59 ± 0.02	1.91 ± 0.02	0.84 ± 0.01
	Spike recovery,[a] %	100	97	102	103	100
Co	Measured conc., μg L^{-1}	$0.02 \pm 0.00_1$	$0.09 \pm 0.00_2$	$0.03 \pm 0.00_{08}$	$0.10 \pm 0.00_4$	$0.11 \pm 0.00_3$
	Conc. after spiking, μg L^{-1}	$0.07 \pm 0.00_2$	$0.14 \pm 0.00_2$	$0.08 \pm 0.00_2$	$0.15 \pm 0.00_4$	$0.16 \pm 0.00_3$
	Spike recovery,[b] %	101	99	102	99	99
Ni	Measured conc., μg L^{-1}	0.58 ± 0.02	1.13 ± 0.02	0.71 ± 0.01	2.43 ± 0.03	0.61 ± 0.02
	Conc. after spiking, μg L^{-1}	1.09 ± 0.03	1.64 ± 0.03	1.22 ± 0.01	2.94 ± 0.03	1.12 ± 0.04
	Spike recovery,[a] %	100	102	101	102	103
Zn	Measured conc., μg L^{-1}	1.13 ± 0.02	0.42 ± 0.01	1.54 ± 0.05	3.15 ± 0.08	12.55 ± 0.19
	Conc. after spiking, μg L^{-1}	1.65 ± 0.04	0.94 ± 0.03	2.04 ± 0.05	3.66 ± 0.08	13.05 ± 0.43
	Spike recovery,[a] %	103	102	101	102	101
Cu	Measured conc., μg L^{-1}	0.19 ± 0.01	$0.22 \pm 0.00_5$	0.72 ± 0.03	1.00 ± 0.01	1.20 ± 0.02
	Conc. after spiking, μg L^{-1}	0.67 ± 0.01	0.72 ± 0.01	1.22 ± 0.03	1.49 ± 0.05	1.70 ± 0.03
	Spike recovery,[a] %	97	99	101	99	100
Cd	Measured conc., μg L^{-1}	$0.03 \pm 0.00_1$	$0.02 \pm 0.00_1$	$0.02 \pm 0.00_{04}$	$0.06 \pm 0.00_1$	$0.08 \pm 0.00_1$
	Conc. after spiking, μg L^{-1}	$0.08 \pm 0.00_1$	$0.07 \pm 0.00_1$	$0.07 \pm 0.00_1$	$0.11 \pm 0.00_5$	$0.13 \pm 0.00_3$
	Spike recovery,[b] %	98	97	99	98	99
Pb	Measured conc., μg L^{-1}	$0.05 \pm 0.00_1$	$0.06 \pm 0.00_{02}$	$0.10 \pm 0.00_1$	0.20 ± 0.01	$0.05 \pm 0.00_2$
	Conc. after spiking, μg L^{-1}	$0.10 \pm 0.00_4$	$0.11 \pm 0.00_1$	$0.15 \pm 0.00_4$	0.24 ± 0.01	$0.10 \pm 0.00_3$
	Spike recovery,[b] %	100	98	101	98	101

[a]: Spiked concentration: 0.5 μg L^{-1}; [b]: spike concentration: 0.05 μg L^{-1}.

Table S6. Operating conditions for the commercial automatic LOV-SPE system

**Monolithic packing**	
Dimension of interlacing cuboids	9.1 mm (L) × 0.9 mm (W) × 0.6 mm (H)
Interstitial distance between cuboids	1.1 mm
Number of cuboids per layer	4 cuboids
Number of layers of interlacing cuboids	40 layers
**SPE scheme**	
Sample condition	pH 8.0
Conditioning buffer	10 mM phosphate buffer
Sample loading flow rate and volume	1.0 mL min^{-1}, 1.4 mL
Evacuation medium	Air
Evacuation flow rate and volume	1.0 mL min^{-1}, 1.4 mL
Eluent	0.5% HNO_3 (v/v)
Elution flow rate and volume	1.0 mL min^{-1}, 4.1 mL
Re-evacuation medium	Air
Re-evacuation flow rate and volume	1.0 mL min^{-1}, 1.4 mL
**ICP-MS**	
ICP mass spectrometer	Agilent 7700x
Plasma forward power	1500 W
Ar gas flow rate	
Plasma gas	15 L min^{-1}
Auxiliary gas	0.9 L min^{-1}
Carrier gas	0.92 L min^{-1}
Makeup gas	0.18 L min^{-1}

Sampling cone	Pt, 1-mm orifice
Skimmer cone	Pt, 0.4-mm orifice
Analysis mode	Time-resolved analysis
Integration time	50 ms
Isotopes monitored	^{55}Mn, ^{59}Co, ^{60}Ni, ^{64}Zn, ^{65}Cu, ^{114}Cd, ^{208}Pb

Table S7. Analytical characteristics of the commercial automatic LOV-SPE system

Element	Working range, ng L^{-1}	Calibration curve	*R*	MDL, ng L^{-1}
^{55}Mn	50–5000	$y = 580[\mathrm{Mn}]^{a} + 32868$	1.0000	1.2
^{59}Co	1–100	$y = 661[\mathrm{Co}]^{a} + 1871$	1.0000	0.4
^{60}Ni	50–5000	$y = 126[\mathrm{Ni}]^{a} + 43719$	1.0000	6.8
^{64}Zn	50–5000	$y = 148[\mathrm{Zn}]^{a} + 36650$	0.9999	5.3
^{65}Cu	50–5000	$y = 220[\mathrm{Cu}]^{a} + 15586$	0.9998	3.0
^{114}Cd	1–100	$y = 502[\mathrm{Cd}]^{a} + 1751$	0.9998	0.3
^{208}Pb	1–100	$y = 233[\mathrm{Pb}]^{a} + 248$	1.0000	1.1

^{a}: ng L^{-1}.

Table S8. Analytical characteristics of the automatic LOV-SPE systems comprising commercial valves with 3D-printed SPE devices and commercial SPE columns for trace metal analysis

Device	Analytes	Sample volume, mL	Throughput, h^{-1}	MDL, ng L^{-1}	Extraction efficiency, %	Capacity, µg cm^{-2}	Reference
4D-printed NIR-actuated LOV-SPE device	Mn, Co, Ni, Cu, Zn, Cd, Pb	1.4	7.2	0.1–6.8	92.7–94.8	63.1–144.9	this study
minicolumn packed with Nobias PA1 resins	Mn, Fe, Co, Ni, Cu, Zn, Cd, Pb	40	0.7	0.01–4.99[a]	–	–	62
3D-printed thiol-functionalized filter scavenger	MeHg and Hg(II)	495	–	0.05–0.08	> 98	–	43
3D-printed right-angled-turned knotted reactor	Mn, Co, Ni, Cu, Zn, Cd, Pb	4.0	15.8	0.1–5.6	73.9–85.9	–	44
SPE column with 4D-printed dual-responsive monolithic packing	Mn, Co, Ni, Cu, Zn, Cd, Pb	0.5	17.1	0.2–7.2	91.9–95.1	51.7–62.7	46
3D-printed column with porous PA6 monolithic packing	Mn, Co, Ni, Cu, Zn, Cd, Pb	1.0	11.5	0.2–7.7	94.3–98.5	27.8–66.4	42
3D-printed column with porous monolithic packing	Mn, Co, Ni, Cu, Zn, Cd, Pb	1.0	12	0.3–6.7	99.2–99.8	5.3–14.3	40

3D-printed preconcentrator	Mn, Ni, Cu, Zn, Cd, Pb	1.0	10	0.3–18.0	–	8.9 (Cu)	36
DLP 3D-printed SPE column with the thermally expanded monolithic foam	Mn, Co, Ni, Cu, Zn, Cd, Pb	0.5	9.0	0.5–5.2	95.6–97.8	77.0–166.8	47
4D-printed temperature-controlled flow-actuated SPE device	Mn, Co, Ni, Cu, Zn, Cd, Pb	5.0	25	0.7–22.1	77.5–96.0	0.85 (Cd)	41
3D-printed SPE column incorporating TiO_2 NP–coated porous monolith	Cr(III), Cr(VI), As(III), As(V), Se(IV), Se(VI)	2.0	15	0.7–32.3	84.5–98.3	45.4–75.9	45
3D-printed minicolumn	Fe(II), Fe(III)	1.0	7.5	1–2	65–87	0.43–2.53	39
Chelex-100 column	V, Mn, Co, Ni, Cu, Zn, As, Cd, Pb	2.0	5.5	5–345	19–100	–	63
3D-printed disk-based SPE device	Cr(VI)	16	6	62.5	–	–	37
3D-printed SPE device	U(VI)	9.0	3	500	93	–	38

[a]: analyzed with magnetic sector inductively coupled plasma mass spectrometry detection
